# Efficacy and safety of electroacupuncture in the treatment of post-stroke cognitive impairment: a systematic review and meta-analysis

**DOI:** 10.3389/fneur.2025.1715658

**Published:** 2026-01-02

**Authors:** Jiayi Zhu, Xihan Ying, Tianqi Huang, Yufei Zhang, Kelin He, Ruijie Ma

**Affiliations:** 1 The Third School of Clinical Medicine (School of Rehabilitation Medicine), Zhejiang Chinese Medical University, Hangzhou, China; 2 The Third Affiliated Hospital of Zhejiang Chinese Medical University (Zhongshan Hospital of Zhejiang Province), Hangzhou, China

**Keywords:** cognitive dysfunction, electroacupuncture, post-stroke cognitive impairment, stroke, systematic review

## Abstract

**Objective:**

The objective of this research was to assess whether electroacupuncture is an effective and safe intervention for post-stroke cognitive impairment (PSCI).

**Methods:**

Our team systematically searched eight academic databases, including the Cochrane Library, PubMed, Embase, Web of Science, China National Knowledge Infrastructure (CNKI), China Biomedical Literature Database (SinoMed), Wanfang Data, and Database of Chinese sci-tech periodicals (VIP). This study conducted a systematic review of randomized controlled trials (RCTs) investigating electroacupuncture for PSCI, covering all available literature from database inception until December 31, 2024. Following a systematic literature screening, data were extracted using Excel. The quality of the included studies was assessed using the Cochrane Risk of Bias tool (RoB 2), and the evidence quality for all outcomes was graded employing the Grading of Recommendations, Assessment, Development, and Evaluations (GRADE) framework. All statistical analyses were performed using R software (version 4.0.0) with the ‘meta’ package. In this study, the Mini-Mental State Examination (MMSE) was used as the primary outcome, while the Montreal Cognitive Assessment (MoCA), the Barthel Index and the Activities of Daily Living (ADL) were used as secondary outcome indicators.

**Results:**

This meta-analysis comprised 24 studies with 1769 patients. The results indicated that after 2 to 8 weeks of electroacupuncture treatment, electroacupuncture was more effective in improving PSCI than the control group (cognitive training, hyperbaric oxygen, western medications, repeated transcranial magnetic stimulation (rTMS), conventional acupuncture, sham electroacupuncture, etc.) and significantly improved post-treatment MMSE (MD = 2.62, 95% CI = 1.74–3.51, *p* < 0.0001, I^2^ = 95.9%), MoCA (MD = 3.01, 95% CI = 2.12–3.91, *p* < 0.0001, I^2^ = 87.0%), Barthel Index (MD = 5.86, 95% CI = 2.71–9.00, *p* = 0.0017, I^2^ = 67.7%), and ADL (MD = 5.82, 95% CI = 0.70–10.94, *p* = 0.0016, I^2^ = 84.4%) scale scores in patients with PSCI. Subgroup analyses indicated that stroke type might be a potential source of heterogeneity for the MMSE and Barthel Index, while treatment duration might contribute to heterogeneity in MoCA scores. Sensitivity analyses revealed that the pooled effect sizes for MMSE, MoCA, and Barthel outcomes remained stable without significant fluctuations, suggesting the robustness of these findings. However, the ADL outcome demonstrated lower robustness. Egger’s test suggested potential publication bias for the MoCA index (*p* = 0.0016).

**Conclusion:**

This systematic review indicates that electroacupuncture may improve cognitive function in patients with PSCI within a short-term period. However, its long-term efficacy and safety profile require further validation through higher-quality evidence. There is a need for future randomized controlled trials with larger sample sizes, longer durations, and more rigorous methodology to verify these findings.

**Systematic review registration:**

PROSPERO registration number CRD420250652626.

## 1 Introduction

Stroke is an acute cerebrovascular disease that results from a sudden reduction or interruption of the blood supply to the brain. This causes the brain tissue to become hypoxic and necrotic, which leads to neurological dysfunction. As the second leading cause of death globally, stroke affects roughly 20% of individuals in high-income countries over their lifetime, while low-income countries face an even higher prevalence (1). Against the backdrop of a large population base and rapid aging in China, the total number of stroke cases and its overall health burden are increasing rapidly (2). Stroke can be categorized into ischemic and hemorrhagic strokes depending on the cause (3), and they can lead to numerous sequelae including cognitive impairment, hemiparesis, and swallowing disorders. Post-stroke cognitive impairment (PSCI) is one of the common sequelae after a stroke, profoundly affecting the survival duration and quality of life of more than 70% of stroke patients (4, 5). PSCI refers to a decline in cognitive abilities emerging within 3 to 6 months after a stroke, affecting one or more areas such as memory, attention, executive function, language, or visuospatial skills (6). Epidemiology estimates that 38% of stroke patients have PSCI during the first year, and that over a five-year period, people with PSCI may have a 61% chance of dying (7, 8). In addition to having a major negative impact on patients’ quality of life, PSCI also places a great deal of strain on family caregivers and dramatically raises the use of social healthcare resources. Cognitive assessment serves as a critical tool for screening PSCI and monitoring treatment efficacy. In clinical practice, the Mini-Mental State Examination (MMSE) and the Montreal Cognitive Assessment (MoCA) are commonly used to screen cognitive function in stroke patients (9). According to guidelines from the European Stroke Organisation (ESO) and the European Academy of Neurology (EAN), both of these instruments are recommended for the cognitive assessment of PSCI (10).

However, the current clinical interventions for PSCI mainly rely on secondary stroke prevention strategies and some Alzheimer’s disease therapeutic drugs, which generally lack strong evidence-based medical support, and there is an urgent need to find new and effective therapeutic modalities to improve the prognosis of patients (11). PSCI is currently treated with a multi-modal comprehensive intervention method. In terms of pharmacological treatment, research have indicated that angiotensin-converting enzyme inhibitors and N-methyl-D-aspartate receptor antagonists are useful in improving cognitive function and patients’ capacity to do everyday tasks, although their side effects still need to be stressed (12). Meanwhile, a variety of drugs, including memantine, ginkgo biloba extract, and some traditional Chinese medicines, also show potential therapeutic value (13–15). It is worth mentioning that there are still no specialized medications for the pharmacological treatment of PSCI, and the clinical evidence for existing drugs is often poor. Non-pharmacological therapies have steadily emerged as a significant area of PSCI research in recent years due to the quick advancements in neuroimaging, artificial intelligence, and neuromodulation technologies. Among these, cognitive rehabilitation training programs focusing on functional compensation and traditional acupuncture therapy have demonstrated promising application prospects (16, 17). It should be noted, although, that the current research still has issues, such as a lack of randomized controlled trials (RCTs) and inconsistent methodological quality, which somewhat compromises the validity and generalizability of the results. To confirm the efficacy of various intervention programs in the future, more excellent clinical research is still required.

The high global incidence of stroke, combined with the limitations of current therapies, makes the integration of acupuncture—which can promote cerebral ischemic tolerance and neuroregeneration—a significant strategy for improving patient outcomes (18). Electroacupuncture therapy, which combines traditional acupuncture theory with current electrical stimulation technology, has demonstrated substantial therapeutic potential in clinical interventions for PSCI due to its benefits of high safety, precise efficacy, and convenient operation (19, 20). Compared to traditional acupuncture, electroacupuncture offers the advantage of combining the effects of needle insertion with electrical pulse stimulation at specific parameters. According to the study, electroacupuncture operates by preventing the proliferation of astrocytes and microglia/macrophages in the peri-infarct regions of the sensorimotor cortex and the P2 purinergic receptor-mediated neuroinflammatory response in the hippocampus CA^1^ area (21). Furthermore, studies have demonstrated that electroacupuncture facilitates microglial M2 polarization via the IL-33/ST2 signaling pathway, enhances white matter structural integrity, and consequently promotes neurological recovery after cerebral ischemia (22). Currently, research on electroacupuncture for PSCI is rapidly developing, but it still has numerous drawbacks that severely limit its clinical and evidentiary quality. For example, treatment methods had significant customized variation, including uneven electroacupuncture parameters, treatment duration, and course length, resulting in low comparability and reproducibility of interventions across trials. Furthermore, most reported RCTs have small sample sizes and low statistical power, making it difficult to detect clinically significant treatment benefits. As a result, completing relevant meta-analyses can not only re-evaluate the clinical efficacy and safety of electroacupuncture for PSCI, but also investigate the influence of various electroacupuncture therapy protocols on cognitive performance.

In this study, we carefully analyzed the aforementioned criteria and systematically investigated the effects of various electroacupuncture stimulation frequencies and treatment durations on cognitive performance in patients with PSCI. This study investigates the efficacy and mechanisms of electroacupuncture in treating PSCI in order to create a more credible experimental foundation for future clinical practice and research approaches.

## 2 Methods

This systematic review and meta-analysis followed the Preferred Reporting Items for Systematic Reviews and Meta-Analyses (PRISMA 2020) guidelines Supplementary material S1 and was registered in the PROSPERO International Prospective Register of Systematic Reviews (Registration ID: CRD420250652626) (23).

### 2.1 Search strategy

Two investigators independently searched the following eight databases: four English-language databases (PubMed, Cochrane Library, Web of Science, and Embase) and four Chinese-language databases [China National Knowledge Infrastructure (CNKI), China Biomedical Literature Database (SinoMed), Wan-fang Database, and VIP Chinese Science and Technology Journal Database (VIP)]. They chose pertinent studies that were published between the databases’ creation and December31, 2024. The search strategy is not restricted by language or region, and it has undergone peer review by experts. Among the search terms used in the database were as follow: “randomized controlled trial,” “stroke,” “cognitive impairment,” and “electroacupuncture.” Specific search algorithms are discussed in Supplementary material S2. When two researchers disagreed on the inclusion of a reference, a third researcher independently determined its eligibility. Additionally, we verified the references cited in the included studies. For a broader review of relevant articles, we will scrutinize references to PSCI-related systematic reviews and systematic evaluations.

### 2.2 Eligibility criteria

#### 2.2.1 Inclusion criteria

The inclusion criteria were established following the PICO (Population, Intervention, Comparison, Outcome) principles.

Study design: RCT. Only published RCTs were included.

Population: This study enrolled patients diagnosed with stroke and comorbid cognitive impairment, with no restrictions regarding age, disease duration, ethnicity, or country. It is important to note that, due to the variability and regional differences in stroke diagnostic criteria, this study explicitly included all studies that employed well-established and clearly defined diagnostic criteria. Cognitive impairment was operationalized as an MMSE score of <27 or a MoCA score of<26 (24).

Interventions: The control group received standard treatment, which included cognitive training, basic care, hyperbaric oxygen therapy, repeated transcranial magnetic stimulation (rTMS), and standardized western medication. The treatment group received additional electroacupuncture therapy in combination with the standard treatment. No restrictions were applied regarding the treatment duration, waveform, frequency, or acupoint selection for the electroacupuncture intervention.

Comparisons: All non-electroacupuncture interventions (including but not limited to cognitive training, medication, rTMS, physical therapy, etc.) were uniformly implemented in both groups, with no restrictions on treatment duration or course.

Outcome: Cognitive function was assessed using the MMSE and the MoCA, with the MMSE serving as the primary outcome measure.; activities of daily living were evaluated using the Barthel Index and the Activities of Daily Living Scale (ADL).

#### 2.2.2 Exclusion criteria

The exclusion criteria comprised the following: (1) Non-randomized controlled trials include retrospective research, reviews, and systematic evaluations, as well as animal experiments and case reports. (2) Studies with ambiguous diagnostic criteria. (3) The study included numerous interventions, and these other measures may have influenced the assessment of electroacupuncture efficacy. (4) Non-stroke-related cognitive impairment. (5) Studies for which the whole text is not available or where the contents are missing.

### 2.3 Study selection

In order to determine the final included literature, we first developed a rigorous censoring process in which two independent researchers (ZJY and HKL) eliminated duplicate papers. Subsequently, we screened study titles and abstracts to eliminate those not meeting our inclusion criteria. Finally, we carefully examined the full text and reviewed all content. A third researcher (YXH) will make the ultimate choice if there is disagreement during the aforementioned procedure.

All database searches and literature curation will be meticulously documented using EndNote Software (version:21), a specialized document management program. Excel was utilized to obtain the following data: total number of participants, gender, age, length of disease, method of intervention, duration of treatment, stroke type, diagnostic criteria, and outcome indicators. If any of the material provided above is incomplete or confusing, we will contact the original author to gather the most comprehensive and correct content possible.

### 2.4 Risk of Bias

To evaluate potential biases in RCTs, we used the risk of bias tool developed by The Cochrane Collaboration (25). We employed the latest Cochrane Risk of Bias assessment tool (RoB 2) to evaluate the methodological quality of the included randomized controlled trials. This tool assesses five key domains—randomization process, deviations from intended interventions, missing outcome data, outcome measurement, and selective reporting of results—through a series of signaling questions (response options include “yes,” “probably yes,” “probably no “,"no” and “no information”). Based on these assessments, the risk of bias for each domain is categorized as “low risk,” “some concerns,” or “high risk.” The evaluation was conducted independently by two investigators (ZJY and HKL). Any disagreements were resolved through consultation with a third investigator (ZYF).

### 2.5 Data synthesis

Meta-analysis, subgroup analysis, publication bias analysis, and sensitivity analysis were all carried out using the “meta” R package (R version 4.0.0). The corresponding 95% CI and mean differences (MD) were computed as impact sizes for the continuous variables MoCA, MMSE, Barthel Index, and ADL. To evaluate heterogeneity, we utilize the I^2^ statistic as the primary indicator, supported by the *p*-value from the Q test for thorough examination. For studies with modest statistical heterogeneity (*p* ≥ 0.05 and I^2^ ≤ 50%), the fixed-effects model is used (26). High heterogeneity (*p* < 0.05 and I^2^ > 50%) warrants sensitivity and subgroup analysis. If the source of heterogeneity remains unknown, the random-effects model is used (27). If heterogeneity cannot be reduced or adequately explained, we will consider abandoning the meta-analysis and instead conduct a qualitative assessment through a systematic descriptive review. We created funnel plots showing the link between effect estimates and their accompanying standard errors in order to evaluate possible publication bias. Utilizing the Egger tests to evaluate the asymmetry of funnel plots.

### 2.6 Data certainty assessment

This study employed the Grading of Recommendations, Assessment, Development, and Evaluations (GRADE) framework to systematically evaluate the quality of evidence for all outcome measures (28). According to the GRADE approach, we assessed the certainty of evidence estimates across five domains: risk of bias, inconsistency, indirectness, imprecision, and publication bias. Based on this comprehensive evaluation, the quality of evidence for each outcome was categorized into one of four levels: “high,” “moderate,” “low,” or “very low.”

## 3 Results

### 3.1 Study selection

**Figure 1:**
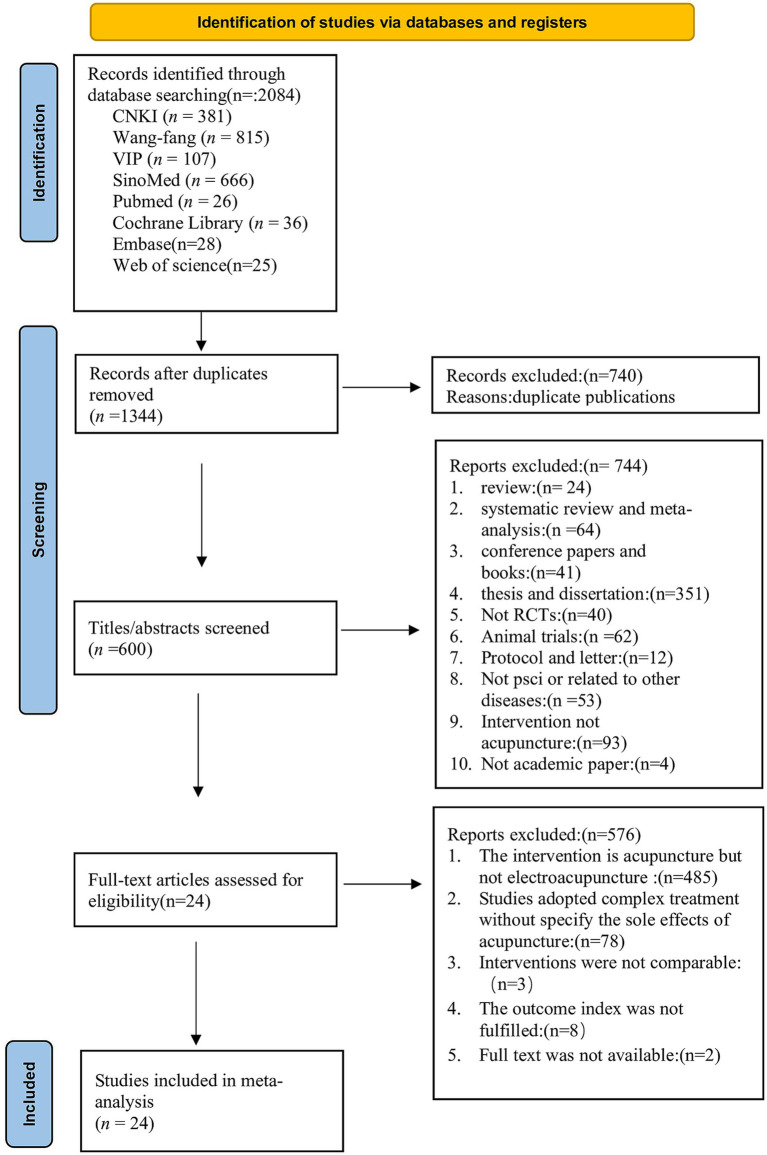
PRISMA flow diagram.

According to the search protocol, a thorough literature search produced 2084 papers, which were dispersed as follows: CNKI (381), VIP (107), Wang-fang (815), SinoMed (666), Web of Science (25), Embase (28), Cochrane Library (29), and PubMed (26). Following the removal of 740 duplicate records and the title/abstract screening of 744 irrelevant studies, 576 full-text publications were subjected to additional review, which eventually led to the inclusion of 24 eligible research (29–52). The flowchart is presented in Figure 1.

### 3.2 Study characteristics

A total of 24 studies were eligible, with 1,769 participants participating, of which 1,034 (58.5%) were male. There were 888 people in the experimental group and 881 in the control group. Baseline data gathered before each RCT was comparable.16 studies (29–32, 36–39, 41, 42, 44–47, 49–51) described the course of the patients’ disease and 8 studies did not describe the course of the disease; all studies reported the duration of treatment, which ranged from 10 days to 8 weeks. One test (40) was a hemorrhagic stroke trial, eight tests (30, 33, 37, 38, 42, 44, 49, 50) were ischemic stroke trials, fifteen tests (29, 31, 32, 34–36, 39, 41, 43, 45–48, 51, 52) were hemorrhagic or ischemic stroke trials. Diagnostic methods in 16 studies included CT or MRI on the basis of expert diagnosis (31–34, 36–40, 43–45, 48–51). Of all the studies, 2 studies used traditional acupuncture as a control group (35, 50), 2 studies used sham electroacupuncture as a control group (30, 48), 2 studies used hyperbaric oxygen as a control group (40, 45), 5 studies used Western medications (Nimodipine, Oxiracetam) as a control group (29, 34, 44, 49, 52), 8 studies used cognitive training as a control group (31–33, 37–39, 41, 47), 2 studies used basal therapy and rTMS as a control group (36, 42), and 3 studies only used basal therapy as a control group (43, 46, 51). Adverse events were documented in 2 studies (30, 48), and no electroacupuncture adverse reactions occurred in either. Trial characteristics are detailed in Table 1.

**Table 1:** Characteristics of 24 studies of cognitive impairment after stroke.

Trial Name	Sample Size		Sex (male/ female)		Age (years)		Disease course (months)		Methods of intervention		Therapy duration (days)	Type of stroke	Acupuncture location	Diagnostic criteria	Outcome measures	Adverse reactions
	T C		T C		T C		T C		T C							
Chen (2013) (35)	30	30	17/13	18/12	66 ± 6	66 ± 7	Not shown		Electroacupuncture + C	Conventional acupuncture	28	Ischemic or hemorrhage stroke	Head + Limbs	Chinese expert consensus standards	MMSE, Barthel	not shown
Feng et al. (2019) (40)	65	69	33/32	35/34	65.2 ± 5.3	63.9 ± 6.8	Not shown		Electroacupuncture + C	Hyperbaric oxygen therapy	40	Hemorrhage stroke	Head	CT or MRI, Chinese expert consensus standards	Barthel	not shown
Gao et al. (2023) (37)	40	40	22/18	23/17	63.5 ± 9.2	63.9 ± 9.3	1.8 ± 0.6	1.9 ± 0.7	Electroacupuncture + C	Basic treatment + cognitive training	42	Ischemic stroke	Head + Limbs	CT or MRI, Chinese expert consensus standards	MMSE, MOCA, Barthel	not shown
Han et al. (2024) (42)	53	53	30/23	29/24	65.33 ± 3.51	65.17 ± 3.61	4.47 ± 0.91	4.32 ± 0.82	Electroacupuncture + C	Basic treatment + rTMS	28	Ischemic stroke	Head + Limbs	Chinese expert consensus standards	MOCA	not shown
Jiao et al. (2024) (30)	16	15	7/9	8/7	61 ± 7	61 ± 4	5.47 ± 1.31	6.02 ± 1.57	Electroacupuncture	Sham Electropuncture	28	Ischemic stroke	Head	Chinese expert consensus standards	MOCA	No adverse reactions
Lei et al. (2021) (36)	31	30	20/11	17/13	58.03 ± 5.53	57.10 ± 5.80	2.2 ± 0.68	2.21 ± 0.70	Electroacupuncture + C	Basic treatment + rTMS	56	Ischemic or hemorrhage stroke	Head	CT or MRI, Chinese expert consensus standards	MOCA, Barthel	not shown
Huang et al. (2021) (48)	60	60	33/27	29/31	65.1 ± 7.5	64.6 ± 8.4	Not shown		Electroacupuncture + C	Sham Electropuncture	56	Ischemic or hemorrhage stroke	Head + Limbs	CT or MRI, Chinese expert consensus standards	MMSE, MOCA, Barthel	No adverse reactions
Li et al. (2023) (65)	30	30	15/15	17/13	65.63 ± 3.82	65.58 ± 3.76	1.41 ± 0.22	1.43 ± 0.25	Electroacupuncture + C	Cognitive training	28	Ischemic or hemorrhage stroke	Head + Limbs	CT or MRI, Chinese expert consensus standards	MMSE, MOCA,	not shown
Li et al. (2021) (49)	30	30	16/14	18/12	60.3 ± 6.2	59.1 ± 7.1	1.20 ± 0.42	1.26 ± 0.33	Electroacupuncture + C	Nimodipine + cognitive training	28	Ischemic stroke	Head + Limbs	CT or MRI, Chinese expert consensus standards	MMSE, MOCA,	not shown
Lin et al. (2009) (50)	28	21	20/8	12/9	65.24 ± 5.30	66.02 ± 5.12	1.29 ± 0.17	1.27 ± 0.16	Electropuncture	Conventional acupuncture	10	Ischemic stroke	Head + Limbs	CT or MRI, Chinese expert consensus standards	MMSE	not shown
Run-li (2017) (43)	32	32	20/12	21/11	56.9 ± 10.3	56.4 ± 10.1	Not shown		Electroacupuncture + C	Basic treatment + cognitive training	14	Ischemic or hemorrhage stroke	Head	CT or MRI, Chinese expert consensus standards	MMSE	not shown
Liu et al. (2013) (41)	25	25	34/16		53.40 ± 8.48		3–12		Electroacupuncture + C	Basic treatment + cognitive training	14	Ischemic or hemorrhage stroke	Head	Chinese expert consensus standards	MMSE	not shown
Ma et al. (2018) (45)	30	30	18/12	16/14	60.97 ± 7.15	60.12 ± 6.56	0.99 ± 0.79	1.04 ± 1.11	Electroacupuncture + C	Hyperbaric oxygen therapy + Oxiracetam	28	Ischemic or hemorrhage stroke	Limbs	CT or MRI, Chinese expert consensus standards	MMSE	not shown
Pan et al. (2023) (38)	45	45	26/19	24/21	66.14 ± 7.28	65.79 ± 6.33	2.37 ± 0.35	2.31 ± 0.49	Electroacupuncture + C	Cognitive training	28	Ischemic stroke	Head	CT or MRI, Chinese expert consensus standard	MMSE, Barthel	not shown
Song et al. (2013) (44)	60	60	39/21	37/23	62.50 ± 4.52	63.01 ± 4.67	0.38 ± 0.14	0.39 ± 0.14	Electroacupuncture + C	Basic treatment + Nimodipine	28	Ischemic stroke	Head	CT or MRI, Chinese expert consensus standard	MMSE	not shown
Sun et al. (2017) (31)	30	30	15/15	15/15	65.07 ± 7.06	64.07 ± 6.58	Not shown		Electroacupuncture + C	Basic treatment + Oxiracetam	56	Ischemic or hemorrhage stroke	Head + Limbs	CT or MRI, Chinese expert consensus standard	MMSE, MOCA, ADL	not shown
Sun et al. (2020) (33)	20	20	8/12	11/9	62.20 ± 12.26	61.75 ± 10.35	Not shown		Electroacupuncture + C	Basic treatment + cognitive training	28	Ischemic stroke	Head + Limbs	CT or MRI, Chinese expert consensus standard	MOCA, Barthel	not shown
Wang et al. (2017) (39)	30	30	20/10	19/11	53.27 ± 11.62	56.73 ± 9.32	3–12		Electroacupuncture + C	Basic treatment + cognitive training	56	Ischemic or hemorrhage stroke	Head + Limbs	CT or MRI, Chinese expert consensus standard	MMSE, ADL	not shown
Wen et al. (2017) (51)	30	30	17/13	18/12	60.2 ± 2.2	59.5 ± 2.7	94.8 ± 21.6	97.2 ± 16.8	Electroacupuncture + C	Basic treatment + cognitive training	28	Ischemic or hemorrhage stroke	Head + Limbs	CT or MRI, Chinese expert consensus standard	MOCA, Barthel	not shown
Yu et al (2020) (11)	37	35	21/16	20/15	65.25 ± 2.79	65.23 ± 2.78	3.42 ± 1.05	3.41 ± 1.03	Electroacupuncture + C	Basic treatment Nimodipine	28	Ischemic or hemorrhage stroke	Head	Chinese expert consensus standard	MMSE, MOCA, ADL	not shown
Zeng et al. (2015) (31)	50	50	30/20	32/18	66 ± 12	68 ± 10	1.15 ± 0.1	1.14 ± 0.1	Electroacupuncture + C	Basic treatment + cognitive training	56	Ischemic or hemorrhage stroke	Head + Limbs	CT or MRI, Chinese expert consensus standard	MOCA, Barthel	not shown
Zhao et al. (2012) (52)	26	26	19/7	28/8	73.35 ± 4.60	72.96 ± 4.95	Not shown		Electroacupuncture	Nimodipine	56	Ischemic or hemorrhage stroke	Head	Chinese expert consensus standard	MMSE	not shown
Zhou et al. (2019) (47)	60	60	36/24	38/22	61.44 ± 8.77	62.04 ± 8.69	5.42 ± 1.87	5.37 ± 1.98	Electroacupuncture + C	Basic treatment + cognitive training	28	Ischemic or hemorrhage stroke	Head	Chinese expert consensus standard	MMSE, MOCA	not shown
Zhou et al. (2013) (29)	30	30	16/14	17/13	64.17 ± 6.43	63.42 ± 5.92	Not shown		Electroacupuncture	Nimodipine	28	Ischemic or hemorrhage stroke	Limbs	Chinese expert consensus standard	MMSE, MOCA	not shown

### 3.3 Risk of bias in studies

**Figure 2:**
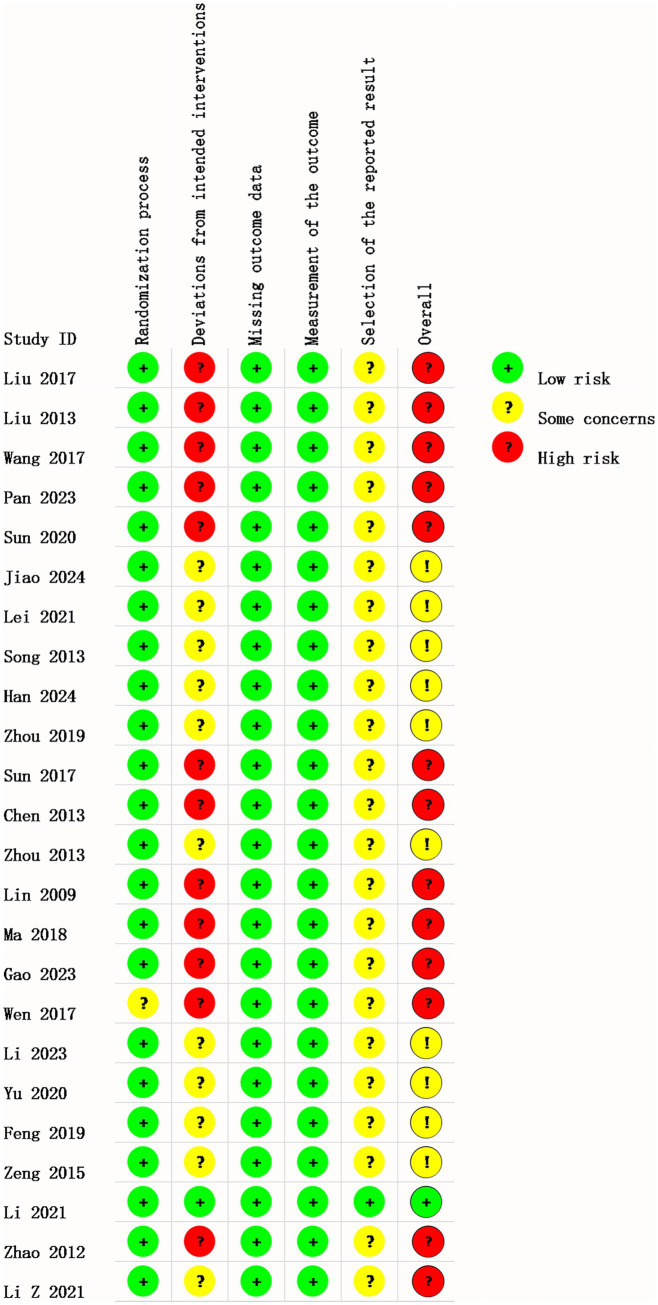
Risk of bias summary: the assessment of each risk of bias item for every included study.

**Figure 3:**
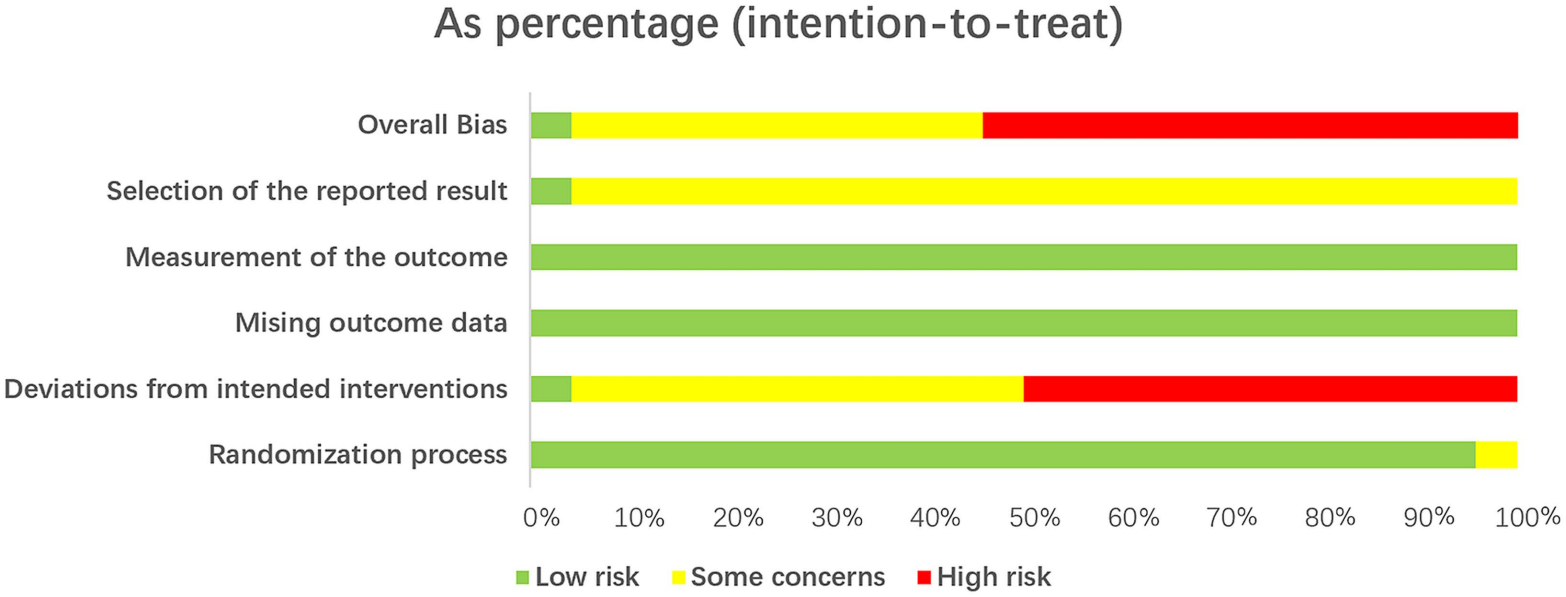
Risk of bias graph: reviewer ratings for each risk of bias item, shown as percentages across all studies.

We assessed the risk of bias for all 24 trials, as shown in Figures 2, 3. The results of the RoB 2 assessment showed that only one study (48) was judged as having a low overall risk of bias, 13 studies (33–35, 37–39, 41, 43, 45, 49–52) (approximately 54%) had a high risk of bias, and the remaining 10 studies (29–32, 36, 40, 42, 44, 46, 47) (approximately 42%) were assessed as having some concerns due to missing key information. Domain-specific assessments revealed the following: in the randomization process domain, 23 studies were rated as low risk as they clearly described the method of random sequence generation, while one study (51) was judged to have some concerns due to an unclear methodology. For deviations from intended interventions, one study (48) was considered low risk due to full adherence to the pre-established protocol, whereas all other studies were rated as having some concerns or high risk because of missing information or unclear implementation. In the domain of missing outcome data, the vast majority of studies were at low risk, supported by complete follow-up or low attrition rates. All studies were rated as low risk in the measurement of the outcome domain, as the outcome assessment was considered appropriate. For selection of the reported result, only one study (48) was classified as low risk due to consistency between the reported results and its registered protocol, while the remaining studies were judged to have some concerns as the original protocols were unavailable.

### 3.4 Meta-analysis of the results

#### 3.4.1 MMSE score

**Figure 4:**
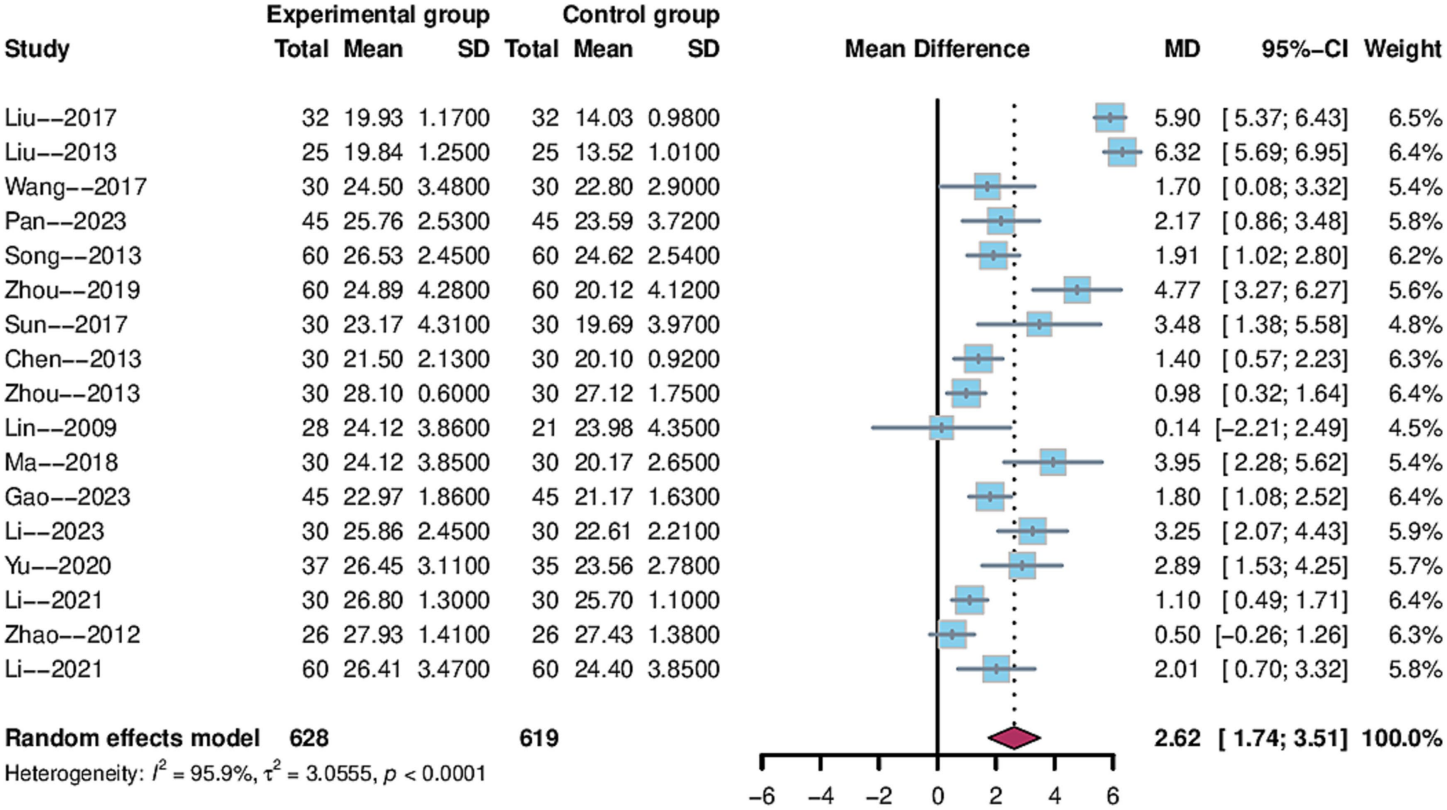
Forest plot of MMSE score.

The MMSE metric was utilized as an outcome indicator in17 trials (29, 32, 34, 35, 37–39, 41, 43–50, 52), with 1,247 patients enrolled, including 628 in the electroacupuncture group and 619 in the control group. The data analysis revealed a 95% confidence interval for MMSE of [1.74, 3.51] and a weighted mean difference of 2.62 (MD = 2.62, 95%CI = 1.74–3.51). The studies showed a high degree of heterogeneity (Heterogeneity: Tau^2^ = 3.05, *p* < 0.0001, I^2^ = 95.9%) using a random effects model (Figure 4).

**Figure 5:**
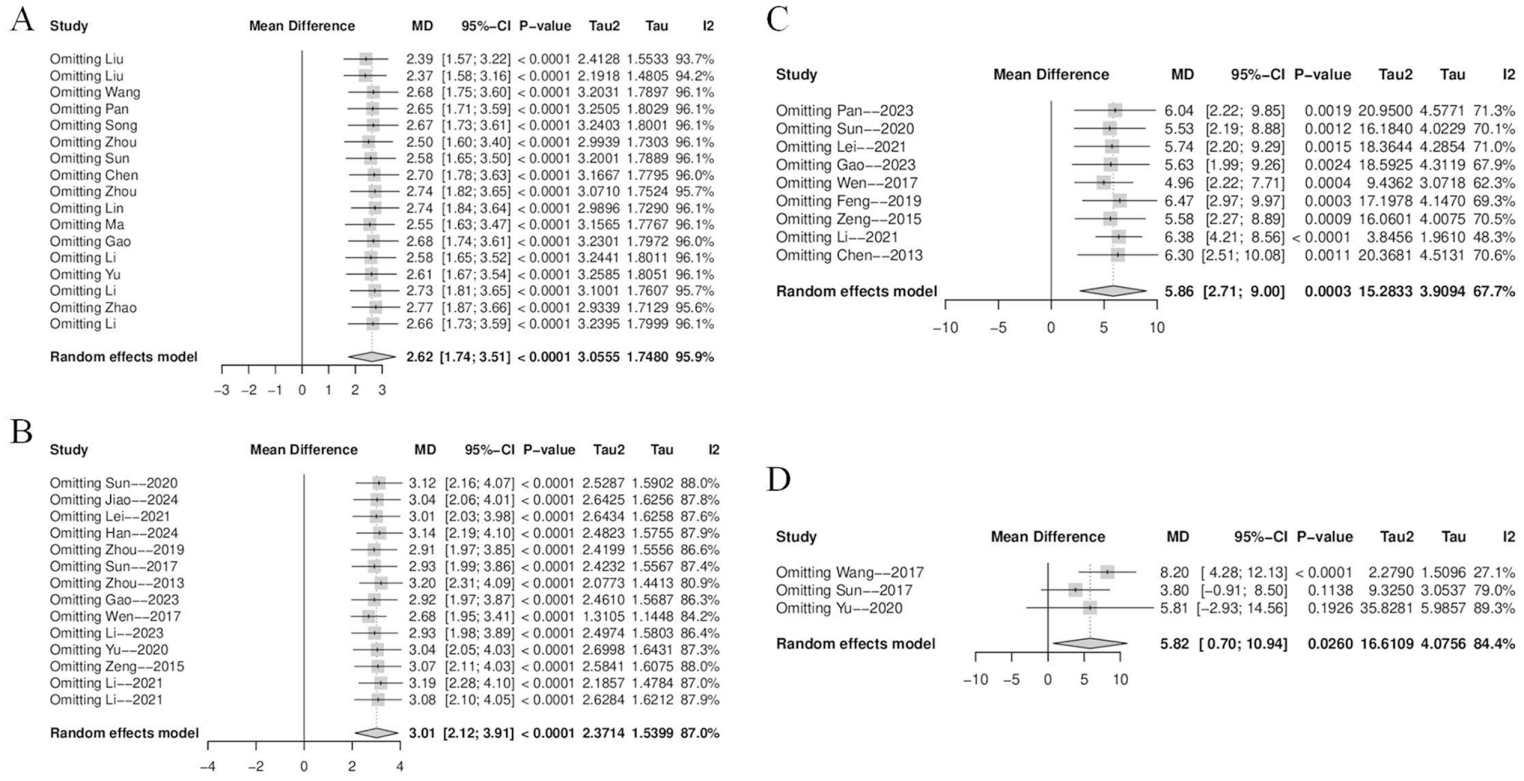
Sensitivity analysis: **(A)** MMSE sensitivity analysis; **(B)** MoCA sensitivity analysis; **(C)** Barthel sensitivity analysis; **(D)** ADL sensitivity analysis.

**Figure 6:**
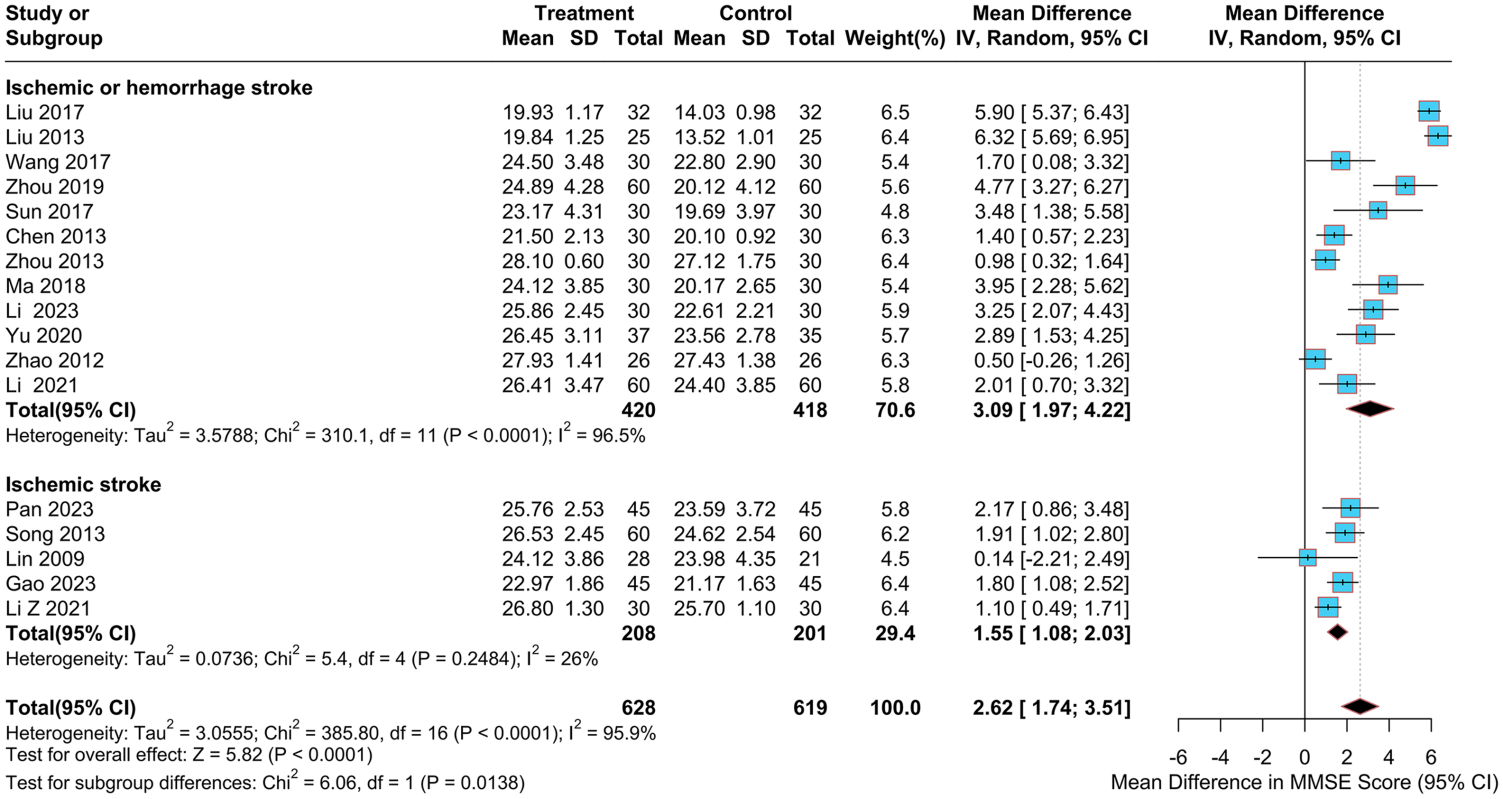
Subgroup analysis of MMSE scores by stroke type.

To identify potential sources of heterogeneity, sensitivity and subgroup analyses were performed. As shown in Figure 5A, the sensitivity analysis confirmed the stability of the pooled results, and sequential exclusion of individual studies did not substantially reduce heterogeneity. Subgroup analyses were conducted for treatment duration, electroacupuncture waveform, stroke type, sample size, control group intervention, acupuncture location and PSCI severity. In the subgroup analysis by stroke type, considerable heterogeneity was observed in the “ischemic or hemorrhagic stroke” subgroup (I^2^ = 96.5%), while heterogeneity was low in the “ischemic stroke” subgroup (I^2^ = 26%), suggesting that stroke type may be a source of heterogeneity (Figure 6). None of the other subgroup analyses showed a significant influence on heterogeneity Supplementary material S3.

#### 3.4.2 MoCA score

**Figure 7:**
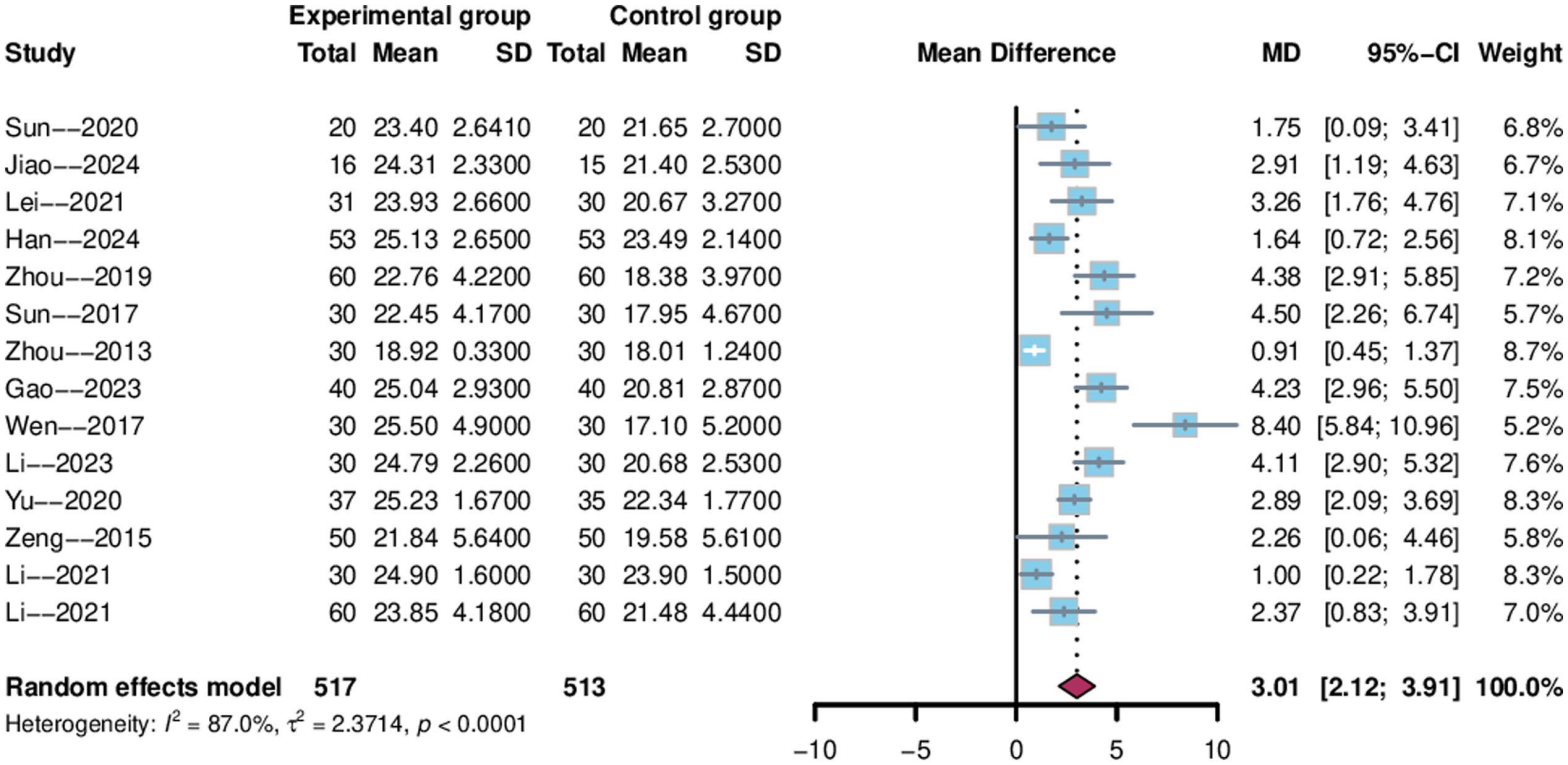
Forest plot of MoCA score.

There were 14 studies (29–34, 36, 37, 42, 46–49, 51) that used MoCA score as an outcome indicator, with a total of 1,030 patients enrolled, 517 in the electroacupuncture group and 513 in the control group. The data analysis revealed a 95% confidence interval of [2.12,3.91] for MoCA and a weighted mean difference of 3.01 (MD = 3.01, 95%CI = 2.12–3.91). The studies showed a high degree of heterogeneity (Heterogeneity: Tau^2^ = 2.37, *p* < 0.0001, I^2^ = 87.0%; Figure 7), so a random effects model was used.

**Figure 8:**
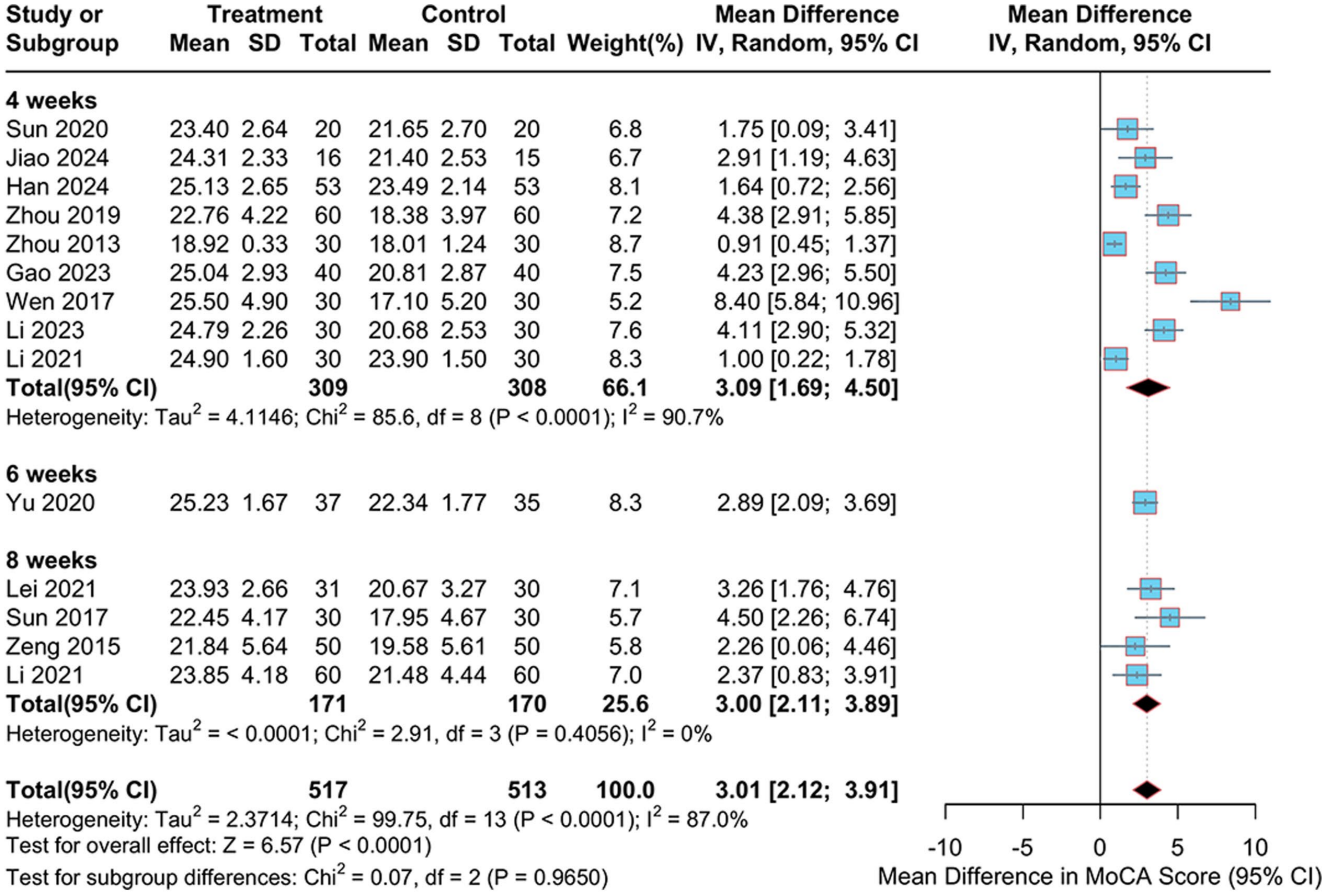
Subgroup analysis of the MoCA scale with different treatment durations.

To explore potential sources of heterogeneity, sensitivity and subgroup analyses were conducted. As shown in Figure 5B, the sensitivity analysis indicated robust and stable results, with no substantial reduction in heterogeneity observed upon sequential removal of individual studies. Subgroup analyses were performed based on treatment duration, electroacupuncture waveform, stroke type, sample size, control group intervention, acupuncture location and severity of PSCI. Analysis by treatment duration revealed considerable heterogeneity in the “4-weeks” subgroup (I^2^ = 90.7%), whereas heterogeneity was low in the “8-weeks” subgroup (I^2^ = 0%), suggesting that treatment duration may be a source of heterogeneity (Figure 8). None of the other subgroup analyses demonstrated a significant impact on heterogeneity (see Supplementary Figure S3).

#### 3.4.3 Barthel score

**Figure 9:**
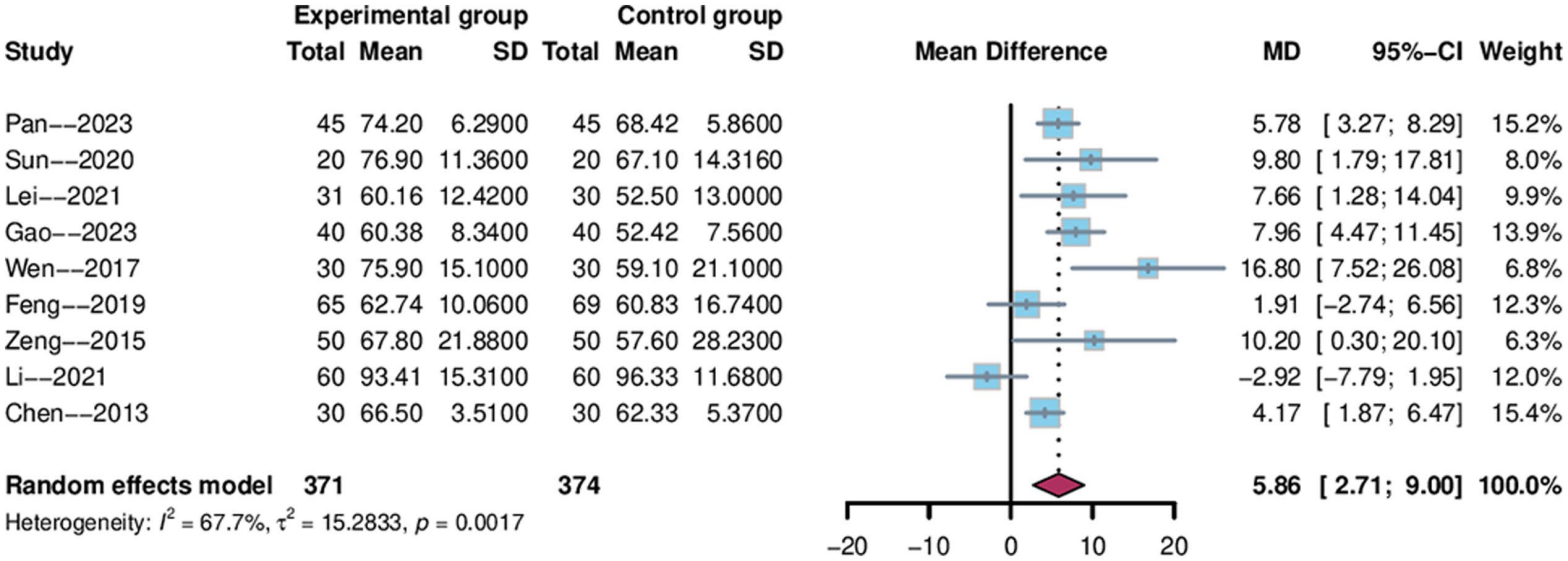
Forest plot of Barthel score.

A total of 9 studies (31, 33, 35–38, 40, 48, 51) used the Barthel index as an outcome indicator, 745 patients were enrolled, with 371 in the electroacupuncture group and 374 in the control group. We employed a random-effects model with a 95% confidence interval for Barthel of [2.71–9.00] and a weighted mean difference of 5.86 (MD = 5.86, 95%CI = 2.71–9.00). The data analysis revealed a high degree of heterogeneity (Heterogeneity: Tau^2^ = 15.2833, *p* = 0.0017, I^2^ = 67.7%; Figure 9), with an I^2^ greater than 50%.

**Figure 10:**
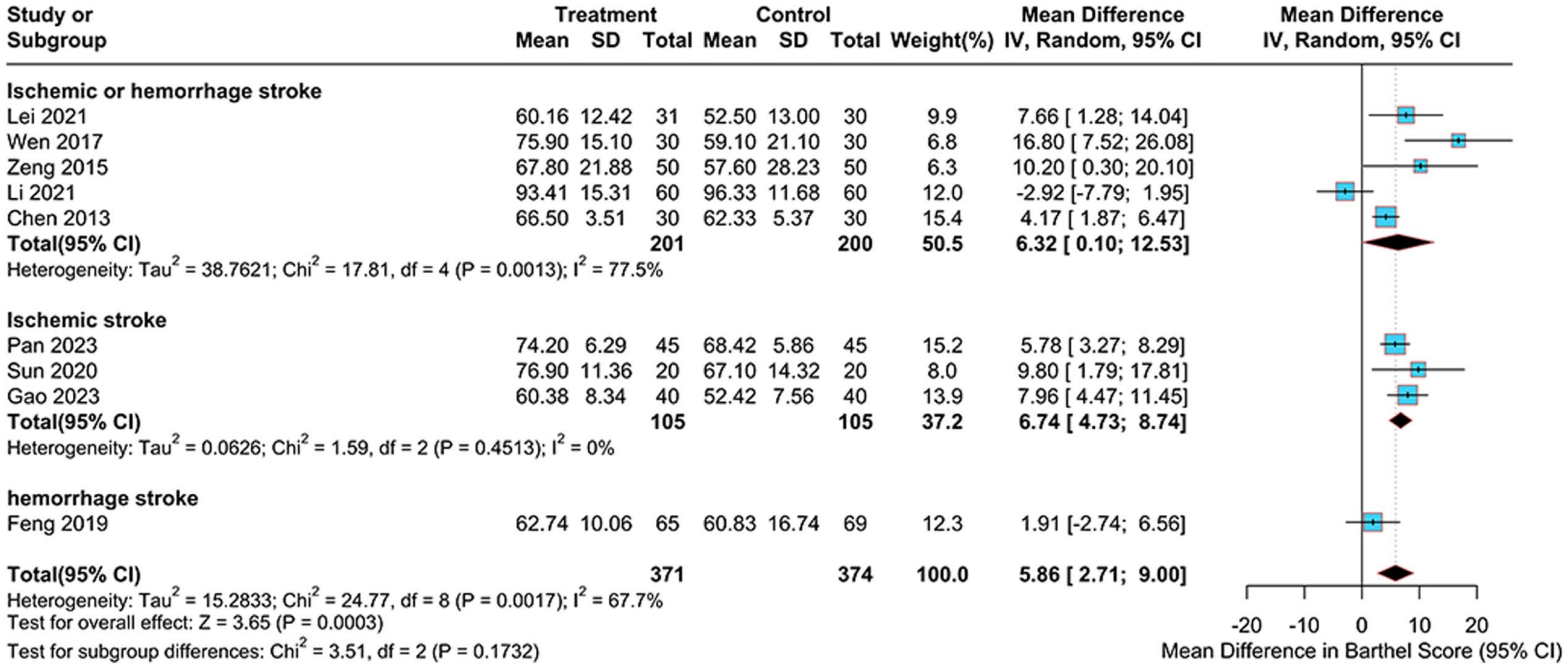
Subgroup analysis of Barthel scores by stroke type.

To identify potential sources of heterogeneity, we conducted both sensitivity and subgroup analyses. Sensitivity analysis revealed that exclusion of the study by Li et al. (48) resulted in reduced heterogeneity (I^2^ decreased from 67.7 to 48.3%), indicating this study might be a source of heterogeneity (Figure 5C). The pooled effect estimate after this exclusion was MD = 6.38 (95% CI: 4.21 to 8.56, *p* < 0.0001). Subgroup analyses were performed based on treatment duration, electroacupuncture waveform, stroke type, sample size, control group intervention, acupuncture location and PSCI severity for the Barthel index. In the stroke type subgroup analysis, considerable heterogeneity was observed in the “ischemic or hemorrhagic stroke” subgroup (I^2^ = 77.5%), while minimal heterogeneity was found in the “ischemic stroke” subgroup (I^2^ = 0%), suggesting that stroke type may be a source of heterogeneity (Figure 10). None of the other subgroup analyses revealed significant effects on heterogeneity (Supplementary Figure S3).

#### 3.4.4 ADL score

**Figure 11:**
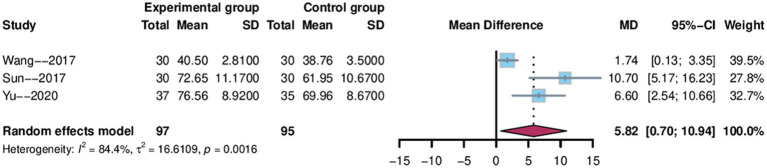
Forest plot of ADL score.

The ADL index was utilized as an outcome indicator in three studies (34, 39, 46), with 192 patients taking part—97 in the electroacupuncture group and 95 in the control group. We analyzed the data using Meta-analysis random effects model, which showed that the 95% confidence interval for ADL was [0.70, 10.94], and the weighted mean difference was 5.82 (MD = 5.82, 95 %CI = 0.70–10.94), and the results of the studies showed a high degree of heterogeneity (Heterogeneity: Tau^2^ = 16.6109, *p* = 0.0016, I^2^ = 84.4%; Figure 11).

We performed a sensitivity analysis and found that after excluding the study by Wang et al. (48), heterogeneity was significantly reduced (I^2^ decreased from 84.4 to 27.1%), indicating that this study may be a major source of heterogeneity (Figure 5D). The pooled effect estimate after exclusion was MD = 8.20 (95% CI: 4.28 to 12.13, *p* < 0.0001). Due to the limited number of studies included, we did not perform subgroup analyses.

### 3.5 Subgroup and sensitivity analysis

The subgroup analyses in this study were conducted across seven dimensions: treatment duration, electroacupuncture waveform, stroke type, sample size, control group intervention, acupuncture location and severity of PSCI. For the sample size subgroup, studies were categorized into large-sample and small-sample groups using the mean sample size (74 cases) of the included studies as the threshold. For the PSCI severity subgroup analysis, this study stratified patients according to the severity of PSCI based on their MMSE or MoCA scores. The specific classification criteria were defined as follows: mild cognitive impairment was indicated by MMSE scores of 21–26 points, moderate by scores of 10–20 points, and severe by scores below 10 points. Corresponding MoCA score ranges were 18–25 points for mild impairment, 10–17 points for moderate impairment, and below 10 points for severe impairment. The subgroup analyses suggested that stroke type may be a potential source of heterogeneity for MMSE and Barthel scores, while treatment duration may contribute to heterogeneity in MoCA scores (Figures 6, 8, 10). We further performed subgroup analyses to assess the impact of different acupoint locations and control interventions on the efficacy of electroacupuncture. Regarding acupoint selection, stimulation of head acupoints yielded the most significant improvements in MMSE and MoCA scores, while the combination of head and limb acupoints was associated with the greatest improvement in the Barthel Index. In terms of control interventions, electroacupuncture was superior to all control types in improving MMSE and MoCA scores, with the most pronounced effects observed when compared to cognitive training. For the Barthel Index, although the greatest effect was also observed versus cognitive training, the results should be interpreted with caution due to the limited number of studies included in some subgroups Supplementary material S3. Results of other subgroup analyses are presented in the Supplementary material S3. Further sensitivity analyses were performed to evaluate the robustness of the results. Sequential exclusion of individual studies demonstrated good robustness for the MMSE, MoCA, and Barthel outcomes, whereas the ADL outcome showed relatively poor robustness (Figure 5). Specifically, heterogeneity in the Barthel index appeared to be primarily attributable to the study by Li et al. (48), while heterogeneity in the ADL outcome may be associated with the study by Wang et al. (39).

### 3.6 Publication bias

**Figure 12:**
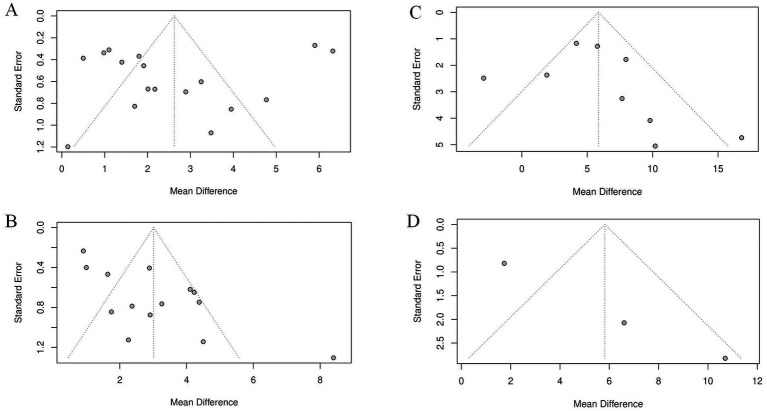
Funnel diagram: **(A)** MMSE funnel diagram; **(B)** MoCA funnel diagram; **(C)** Barthel funnel diagram; **(D)** ADL funnel diagram.

To systematically assess potential publication bias, we conducted both funnel plot analysis and Egger’s test for the MMSE, MoCA, Barthel and ADL indices (Figure 12). The funnel plots for all four outcomes showed some asymmetry, suggesting possible publication bias. However, the Egger’s test results were inconsistent: no significant publication bias was detected for the MMSE (t = −0.76, df = 15, *p* = 0.457) or Barthel index (t = 0.88, df = 7, *p* = 0.4098), whereas a statistically significant bias was identified for the MoCA (t = 4.04, df = 12, *p* = 0.0016).

The significant publication bias observed for the MoCA outcome may be attributed to several factors. First, the prevalent preference for publishing positive results may have led to selective publication of studies demonstrating favorable outcomes. Second, most included studies had small sample sizes (n < 70), which are inherently underpowered and more susceptible to such publication preferences. Additionally, the majority of studies were conducted in China, where high homogeneity among study populations might further discourage the publication of negative results that lack novelty. Finally, as the MoCA is a sensitive tool for detecting cognitive changes, it may be preferentially used in studies aiming to demonstrate therapeutic efficacy. These factors collectively contribute to the observed publication bias.

### 3.7 Safety of electroacupuncture

Among the 24 randomized controlled trials included in this systematic review, only two (30, 48) (8.3%) explicitly documented adverse events related to electroacupuncture, with no significant side effects reported in either study. The remaining 22 studies (91.7%) did not provide any safety-related information. Although the limited data available did not indicate major safety concerns, the overall evidence remains insufficient to support definitive conclusions on the safety of electroacupuncture for PSCI, primarily due to the lack of systematic safety reporting in the majority of included studies.

### 3.8 GRADE assessment

**Figure 13:**
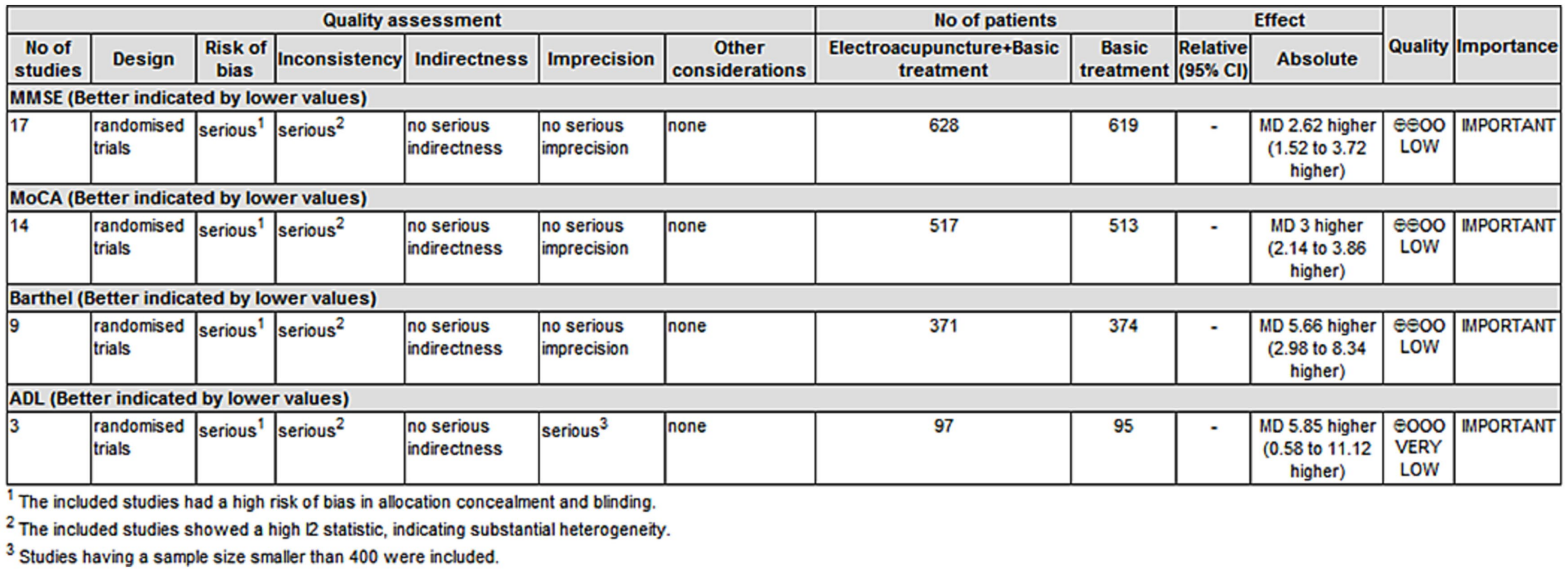
Summary of evidence quality for outcome measures (MMSE, MoCA, Barthel Index, and ADL).

The evidence quality for MMSE, MoCA, and Barthel scores was rated as “low” according to the GRADE criteria. This rating was based on a “serious” risk of bias, as most studies demonstrated high risk in allocation concealment and blinding implementation, along with “serious” inconsistency due to considerable heterogeneity (I^2^ > 50%). The evidence quality for the ADL score was further downgraded to “very low,” owing to the “serious” risk of bias and “serious” inconsistency as mentioned above, in addition to “serious” imprecision resulting from a total sample size of fewer than 400 participants (Figure 13).

## 4 Discussion

Cognitive impairment, a typical consequence following stroke, has a substantial impact on patients’ rehabilitation and social function reconstruction (53). The pathogenesis of PSCI stems from focal cerebral structural damage triggered by ischemic or hemorrhagic stroke and further leads to neural network dysfunction. Although the pathophysiologic processes of the disease are not completely known, available information suggests that its development may be connected with a cascade of pathologic responses caused by acute stroke and is frequently based on preexisting microangiopathy and neurodegeneration (54). Potential biomarkers of PSCI cover multimodal indicators, including neuroimaging features humoral metabolomic alterations, genetic susceptibility markers, and systemic inflammatory mediators (55). PSCI is currently treated with a multifaceted approach that includes pharmacological interventions (cholinesterase inhibitors or NMDA receptor antagonists may improve cognition, although the evidence is limited; some patients may try nimodipine or ginkgo biloba), non-pharmacological therapies (such as cognitive rehabilitation and lifestyle modification), and etiological treatment (control of vascular risk factors like hypertension and diabetes, and use of antiplatelet or anticoagulant therapy to prevent recurrent strokes). There are no particular treatments available; instead, the focus is on personalized care and early prevention. Electroacupuncture, an essential therapy in Chinese medicine, has distinct benefits in the treatment of stroke, peripheral nerve injury, and neurodegenerative illnesses. This interventional modulation more effectively suppresses the nervous system’s stress response and corrects functional imbalances between the brain’s excitatory and inhibitory systems by regulating neural electrophysiological activity (56). Furthermore, by regulating neurotransmitter release, promoting synaptic plasticity, and activating brain functional network reorganization, electroacupuncture not only improves motor, sensory, and cognitive dysfunction, but also enhances neural repair and functional compensation, indicating a broad application potential in the field of neurorehabilitation. It has been suggested that electroacupuncture may promote mitochondrial fusion and inhibit fission by down-regulating the expression of the SIRT1/PGC-1α signaling pathway, thereby attenuating hippocampal neuronal damage, which may be the mechanism of its action to ameliorate the cognitive dysfunction in the MCAO/R model rats (57). Another study demonstrated that electroacupuncture ameliorates cognitive impairment and neurological damage in rats with ischemic stroke by regulating the MEG3/miR-4640-3p axis (58). Electroacupuncture can improve memory function in ischemic stroke model rats by down-regulating the expression of P2X7 receptor (P2X7R), attenuating the inflammatory response and decreasing the reactive oxygen species (ROS) level, which provides a potential strategy for ischemic stroke treatment targeting P2X7R as an intervention target (59). The findings of this study suggest that electroacupuncture is useful in treating cognitive deficits in stroke patients, even if more research is required to identify the most effective clinical use of electroacupuncture for treating PSCI.

In evaluating the efficacy of electroacupuncture for PSCI, we observed that although all outcome measures (including MMSE, MoCA, Barthel Index, and ADL) suggested potential benefits of electroacupuncture in improving cognitive function and activities of daily living in PSCI patients, these results were accompanied by substantial heterogeneity, warranting cautious interpretation. To explore the sources of heterogeneity and assess the robustness of the findings, we performed sensitivity and subgroup analyses. Sensitivity analysis indicated that the results for MMSE, MoCA, and the Barthel Index remained robust, whereas the ADL outcomes were less reliable due to the limited number of included studies and considerable influence from the study by Wang et al. (2017). Therefore, conclusions regarding the effect of electroacupuncture on improving activities of daily living in PSCI patients should be interpreted with considerable caution. Subgroup analysis suggested that stroke type may be a potential source of heterogeneity for MMSE and the Barthel Index, while treatment duration might contribute to heterogeneity in MoCA outcomes. Nevertheless, given the consistently high heterogeneity across outcomes, other potential sources of heterogeneity were also considered. First, there were significant heterogeneity in the therapies themselves. Inconsistencies in electroacupuncture parameters (e.g., waveform), treatment length, acupoint selection processes, and operator techniques and expertise can all have a major impact on efficacy ratings, making them the primary source of variability. For instance, studies have demonstrated that acupuncture at different acupoints activates distinct neural pathways. These pathways integrate at multiple levels, including the spinal cord, brainstem, and cerebral cortex, resulting in synergistic or antagonistic effects, which explains why combinations of different acupoints lead to divergent therapeutic outcomes (60). Functional magnetic resonance imaging (fMRI) studies have revealed that variations in needle manipulation techniques among acupuncturists can elicit “deqi” sensations of varying intensities. The strength of these sensations is significantly correlated with the degree of activation in specific brain regions, indicating that the practitioner’s technique can influence therapeutic efficacy by modulating responses within the central nervous system (61). Second, baseline features of patients with PSCI differed between research, including the severity of brain functional abnormalities as well as the location and extent of infarction or hemorrhage sites. These pathophysiological traits may influence therapeutic results, resulting in heterogeneity (62). Furthermore, this study integrated several control interventions into a single “control group.” While this strategy improved statistical efficiency, the underlying disparities in effects caused by different control measures may have increased the degree of heterogeneity. To conclude, the current evidence suggests potential benefits of electroacupuncture for cognitive function in PSCI; however, any conclusion regarding the improvement of activities of daily living must be approached with caution, given the considerable heterogeneity and limited number of available studies.

Safety is a critical consideration in electroacupuncture therapy for PSCI. However, in this systematic review, only two (8.3%) of the included randomized controlled trials explicitly documented adverse events related to electroacupuncture, with neither reporting any significant side effects. The current body of evidence remains insufficient to conclusively establish the safety of electroacupuncture for PSCI. Future high-quality studies incorporating rigorous safety monitoring are warranted to confirm its safety profile in PSCI patients. To enhance the quality and standardization of safety reporting, it is strongly recommended that researchers strictly adhere to the adverse event reporting checklist outlined in the Consolidated Standards of Reporting Trials (CONSORT 2025 statement) (63). This entails the systematic documentation of event type (e.g., subcutaneous hematoma, fainting during acupuncture), severity grading (mild, moderate, severe), and its causal relationship to the electroacupuncture intervention.

This systematic review differs from previous studies in several key aspects. Compared with the study by Yi et al. (2024), which focused on patients with vascular dementia—a dementia syndrome caused by chronic cerebrovascular pathology—this study targets PSCI, emphasizing cognitive deficits occurring within 3–6 months after an acute stroke, which aligns more closely with the clinical needs of stroke rehabilitation. In terms of interventions, Yi et al. (64) comparing 21 non-pharmacological interventions (such as acupuncture combined with moxibustion, rehabilitation training, and rTMS) but did not explore specific intervention parameters in depth. In contrast, this study specifically evaluates electroacupuncture and documents detailed parameters (e.g., waveform, frequency, treatment duration). Compared with the study by Li et al. (65), which assessed the methodological quality of 14 systematic reviews on acupuncture for PSCI in the form of an overview, our study is the first to conduct a systematic review and meta-analysis specifically on electroacupuncture for PSCI. By directly synthesizing original data from 24 RCTs involving 1,769 patients, and applying Cochrane RoB 2 and GRADE tools to assess bias risk and evidence quality, this study provides new evidence on the efficacy and safety of electroacupuncture. Furthermore, through subgroup and sensitivity analyses, this study explores sources of heterogeneity and offers insights for optimizing electroacupuncture treatment protocols, addressing a gap in the standardization of electroacupuncture parameters noted in Li et al. (65).

### 4.1 Limitations and recommendations for future research

#### 4.1.1 Limitations to generalization

No language restrictions were applied during the search; however, no relevant publications in languages other than Chinese and English were identified. Among the 24 included studies, 23 were published in Chinese, and only one was in English, indicating that the majority of the RCTs were conducted in China. This distribution aligns closely with the global trend highlighted in the bibliometric analysis by Zhang et al. (66), which identified China as the dominant contributor to research on acupuncture for stroke. Consequently, the validity and applicability of our findings to non-Chinese populations and different healthcare systems—particularly non-Traditional Chinese Medicine (TCM) dominated rehabilitation settings—remain unclear. This may be closely related to the fact that electroacupuncture is deeply rooted in TCM theory and practice. For instance, the intervention protocols used in the included studies—such as the selection of specific acupoints [e.g., Baihui (GV20), Sishencong (EX-HN1)] and the operators’ emphasis on achieving deqi—are derived from TCM theory. We acknowledge the limited generalizability of these results. There is an urgent need for future high-quality RCTs that include multi-ethnic and multi-regional samples. These studies should be designed to account for the influences of cultural adaptation and healthcare system differences on intervention effects, thereby systematically examining the generalizability of electroacupuncture across diverse populations and clinical settings.

#### 4.1.2 Limited quality of evidence

The methodological quality of the 24 clinical trials included in the analysis was compromised by several limitations. Specifically, 83% of the trials did not discuss or implement allocation concealment, with only a few potentially adopting necessary precautions without reporting them. Regarding blinding, the implementation of double-blinding was particularly challenging due to the nature of electroacupuncture intervention. Only one study employed a double-blind design; the lack of blinding in others may have undermined the objectivity of outcome assessment. Consequently, according to the GRADE criteria, the quality of evidence for the MMSE, MoCA, and Barthel Index was rated as “low,” while that for the ADL was rated as “very low.” Collectively, these limitations constrain the overall quality of the included studies and may affect the reliability of the conclusions.

Therefore, future large-scale, high-quality RCTs are warranted to validate the existing findings. Firstly, reliable sham electroacupuncture should be adopted as a control to facilitate double-blinding. Secondly, stroke types should be specified in the inclusion criteria, as subgroup analysis suggested that ischemic stroke patients showed more significant improvements in MMSE and Barthel Index with lower heterogeneity (MMSE: I^2^ = 26%; Barthel: I^2^ = 0%). Additionally, the treatment protocol should prioritize commonly used acupoints with established evidence-based support [e.g., Baihui (GV20), Sishencong (EX-HN1), Zusanli (ST36)], and incorporate foundational research to determine optimal electroacupuncture frequency and waveform parameters. Finally, a treatment duration of ≥8 weeks is recommended, as this period was associated with significant MoCA improvement and low heterogeneity (I^2^ = 0%). Long-term follow-up should also be implemented to evaluate the sustained therapeutic effects in the chronic management of PSCI.

#### 4.1.3 Limitation of short treatment duration

As PSCI is a chronic and progressive condition, the short intervention and follow-up durations (≤2 months) in the included studies prevent an evaluation of the sustained efficacy of electroacupuncture and its long-term value in dementia prevention. Therefore, future research must incorporate long-term follow-ups (e.g., at 6 and 12 months post-treatment) to determine whether electroacupuncture can maintain cognitive benefits and delay disease progression, thereby providing evidence for its role in the long-term management of PSCI.

In conclusion, future large-scale, high-quality RCTs are warranted to validate the current findings. Furthermore, it is essential to explore the optimal stimulation parameters for electroacupuncture in treating PSCI—such as waveform, frequency, current intensity, and treatment duration—to optimize therapeutic protocols and enhance efficacy. Finally, integrating neuroimaging studies with basic experimental research could provide deeper insights into the mechanisms of electroacupuncture for PSCI, thereby establishing a more robust theoretical foundation for clinical practice.

## 5 Conclusion

This systematic review indicates that electroacupuncture may improve cognitive function in patients with PSCI within a short-term period (2–8 weeks). However, its long-term efficacy (>8 weeks) and safety profile require further validation through higher-quality evidence. Due to the generally low quality of the included trials and limitations related to regional and cultural factors, the generalizability of the current findings is limited and may only be applicable to Chinese populations. Future research should focus on standardizing electroacupuncture procedures and treatment protocols, while conducting large-sample, long-term follow-up, and rigorously blinded randomized controlled trials to generate more universally applicable evidence.

## Data Availability

The original contributions presented in the study are included in the article/supplementary material, further inquiries can be directed to the corresponding author.

## References

[1] Hilkens NA Casolla B Leung TW de Leeuw FE. Stroke. Lancet. (2024) 403:2820–36. doi: 10.1016/s0140-6736(24)00642-1, 38759664

[2] Ji C Ge X Zhang J Tong H. The stroke burden in China and its long-term trends: insights from the global burden of disease (Gbd) study 1990-2021. Nutr Metab Cardiovasc Dis. (2025) 35:103848. doi: 10.1016/j.numecd.2025.103848, 39948019

[3] Walter K. What is acute ischemic stroke? JAMA. (2022) 327:885. doi: 10.1001/jama.2022.1420, 35230392

[4] Lawson TN Balas MC McNett M. A scoping review of the incidence, predictors, and outcomes of delirium among critically ill stroke patients. J Neurosci Nurs. (2022) 54:116–23. doi: 10.1097/jnn.0000000000000642, 35532330

[5] Li ZT Ban LQ Chen F. Acupuncture of revised Acupoint combination around the Skull Base for post-stroke mild cognitive impairment: a randomized controlled trial. Zhongguo Zhen Jiu. (2023) 43:1104–8. doi: 10.13703/j.0255-2930.20221231-0002, 37802513

[6] Jokinen H Melkas S Ylikoski R Pohjasvaara T Kaste M Erkinjuntti T . Post-stroke cognitive impairment is common even after successful clinical recovery. Eur J Neurol. (2015) 22:1288–94. doi: 10.1111/ene.12743, 26040251

[7] Lo JW Crawford JD Desmond DW Godefroy O Jokinen H Mahinrad S . Profile of and risk factors for poststroke cognitive impairment in diverse ethnoregional groups. Neurology. (2019) 93:e2257–71. doi: 10.1212/WNL.0000000000008612, 31712368 PMC6937495

[8] Sexton E McLoughlin A Williams DJ Merriman NA Donnelly N Rohde D . Systematic review and meta-analysis of the prevalence of cognitive impairment no dementia in the first year post-stroke. Eur Stroke J. (2019) 4:160–71. doi: 10.1177/2396987318825484, 31259264 PMC6591758

[9] Nasreddine ZS Phillips NA Bédirian V Charbonneau S Whitehead V Collin I . The Montreal cognitive assessment, Moca: a brief screening tool for mild cognitive impairment. J Am Geriatr Soc. (2005) 53:695–9. doi: 10.1111/j.1532-5415.2005.53221.x, 15817019

[10] Quinn TJ Richard E Teuschl Y Gattringer T Hafdi M O'Brien JT . European stroke organisation and European academy of neurology joint guidelines on post-stroke cognitive impairment. Eur Stroke J. (2021) 6:I–XXXVIII. doi: 10.1177/23969873211042192, 34746430 PMC8564156

[11] Yu KH Cho SJ Oh MS Jung S Lee JH Shin JH . Cognitive impairment evaluated with vascular cognitive impairment harmonization standards in a multicenter prospective stroke cohort in Korea. Stroke. (2013) 44:786–8. doi: 10.1161/STROKEAHA.112.668343, 23271507

[12] Li Y Cui R Liu S Qin Z Sun W Cheng Y . The efficacy and safety of post-stroke cognitive impairment therapies: an umbrella review. Front Pharmacol. (2023) 14:1207075. doi: 10.3389/fphar.2023.1207075, 37693907 PMC10483224

[13] McShane R Westby MJ Roberts E Minakaran N Schneider L Farrimond LE . Memantine for dementia. Cochrane Database Syst Rev. (2019) 3:Cd003154. doi: 10.1002/14651858.CD003154.pub6 30891742 PMC6425228

[14] Kandiah N Ong PA Yuda T Ng LL Mamun K Merchant RA . Treatment of dementia and mild cognitive impairment with or without cerebrovascular disease: expert consensus on the use of *Ginkgo biloba* extract, Egb 761(®). CNS Neurosci Ther. (2019) 25:288–98. doi: 10.1111/cns.13095, 30648358 PMC6488894

[15] Pennington E Bell S Hill JE. Should video laryngoscopy or direct laryngoscopy be used for adults undergoing endotracheal intubation in the pre-hospital setting? A critical appraisal of a systematic review. J Paramed Pract. (2023) 15:255–9. doi: 10.1002/14651858, 38812899 PMC7616025

[16] Quinn TJ Richard E Teuschl Y Gattringer T Hafdi M O'Brien JT . European stroke organisation and European academy of neurology joint guidelines on post-stroke cognitive impairment. Eur J Neurol. (2021) 28:3883–920. doi: 10.1111/ene.15068 34476868

[17] Chen X Zhang L Mao W Wen J Wang Y Ma K . Comparing the effect of acupuncture, sham acupuncture, and waiting-list control on patients with post-stroke cognitive impairment: a randomized clinical trial. QJM. (2025). doi: 10.1093/qjmed/hcaf181 40971519

[18] Liu X Qian Z Li Y Wang Y Zhang Y Zhang Y . Unveiling synergies: integrating Tcm herbal medicine and acupuncture with conventional approaches in stroke management. Neuroscience. (2025) 567:109–22. doi: 10.1016/j.neuroscience.2024.12.043, 39730019

[19] Li K Xu S Wang R Zou X Liu H Fan C . Electroacupuncture for motor dysfunction and constipation in patients with Parkinson's disease: a randomised controlled multi-Centre trial. EClinicalMedicine. (2023) 56:101814. doi: 10.1016/j.eclinm.2022.101814, 36691434 PMC9860357

[20] Guo Y Sun T Qiu F Li X Cui W Liao Z . Electroacupuncture combined with cognitive rehabilitation outperforms cognitive rehabilitation alone in treating post-stroke cognitive impairment: a randomized controlled trial. Front Neurol. (2025) 16:1507475. doi: 10.3389/fneur.2025.1507475, 39944540 PMC11814160

[21] Huang J You X Liu W Song C Lin X Zhang X . Electroacupuncture ameliorating post-stroke cognitive impairments via inhibition of peri-infarct astroglial and microglial/macrophage P2 purinoceptors-mediated neuroinflammation and hyperplasia. BMC Complement Altern Med. (2017) 17:480. doi: 10.1186/s12906-017-1974-y, 29017492 PMC5635586

[22] Zhang Y Ding Z Wang Y Liu H Gao J Wang H . Electroacupuncture protects against post-stroke cognitive impairment by promoting an Il-33/St2 axis-mediated microglia M2 polarization. Neurochem Res. (2025) 50:261. doi: 10.1007/s11064-025-04522-8, 40801996

[23] Page MJ McKenzie JE Bossuyt PM Boutron I Hoffmann TC Mulrow CD . The Prisma 2020 statement: an updated guideline for reporting systematic reviews. BMJ. (2021) 372:n71. doi: 10.1136/bmj.n71, 33782057 PMC8005924

[24] Chun CT Seward K Patterson A Melton A MacDonald-Wicks L. Evaluation of available cognitive tools used to measure mild cognitive decline: a scoping review. Nutrients. (2021) 13. doi: 10.3390/nu13113974, 34836228 PMC8623828

[25] Higgins JP Altman DG Gøtzsche PC Jüni P Moher D Oxman AD . The Cochrane collaboration's tool for assessing risk of bias in randomised trials. BMJ. (2011) 343:d5928. doi: 10.1136/bmj.d5928, 22008217 PMC3196245

[26] Mantel N Haenszel W. Statistical aspects of the analysis of data from retrospective studies of disease. J Natl Cancer Inst. (1959) 22:719–48. 13655060

[27] DerSimonian R Laird N. Meta-analysis in clinical trials revisited. Contemp Clin Trials. (2015) 45:139–45. doi: 10.1016/j.cct.2015.09.002, 26343745 PMC4639420

[28] Schünemann HJ Brennan S Akl EA Hultcrantz M Alonso-Coello P Xia J . The development methods of official grade articles and requirements for claiming the use of grade - a statement by the grade guidance group. J Clin Epidemiol. (2023) 159:79–84. doi: 10.1016/j.jclinepi.2023.05.010, 37211327

[29] Zhou THP Cheng H. Clinical study on the treatment of vascular cognitive impairment with electroacupuncture related well points. Clin J Tradit Chin Med. (2013) 8

[30] Jiao M Wang D He L Feng L Wang R Sui X. Brian effects of Electroacupuncture on sleep quality and cognitive function in patients with insomnia related to cerebral infarction. Zhongguo Zhen Jiu. (2024) 44:1107–13. doi: 10.13703/j.0255-2930.20230703-k0004, 39401806

[31] Zeng Y Bao Y Zhu M Chen S Fang J. Mild cognitive impairment of stroke at subacute stage treated with acupuncture: a randomized controlled trial. Zhongguo Zhen Jiu. (2015) 35:979–82. doi: 10.13703/j.0255-2930.2015.10.001 26790200

[32] Li Leishen QJ SUN Y HUANG G. Effect of cognitive rehabilitation training combined with acupoint electroacupuncture on patients with post - stroke cognitive impairment. Henan Med Res. (2023) 32: 3508–12. doi: 10.3969/j.issn.1004-437X.2023.19.012

[33] Sun Qing LJ Cheng X Su Q Chen X Lu j Gao Y . Effects of electroacupuncture on attention and daily living skills in patients with nondemented vascular cognitive impairment. Chin J Geriat Care. (2020) 18:27–30. doi: 10.3969/j.issn.1672-2671.2020.01.009

[34] SUN Shanbin CE CHEN C HAO P SUN X YANG Y LI Y . Clinical observation of Shenting and Sishencong acupuncture with the dialectical acupoints in treating cognitive dysfunction after stroke. Clin J Tradit Chin Med. (2017) 29

[35] CHEN Ying-Hua SZ-R DU W-X NI G-z Jiang L Hong X Qin R-q . Clinical study of electroacupuncture at points Sishencong and Fengchi for the treatment of vascular dementia. Shanghai J Acupunct Moxibustion. (2013) 32: 245–7. Epub 20130903. doi: 10.3969/j.issn.1005-0957.2013.04.245

[36] Lei Xiaoling DY Chanjuan Z Linling D Qixuan S. Electroacupuncture combined with repetitive transcranial magnetic stimulation in the treatment of post stroke cognitive impairment. Acta Chin Med. (2021) 36:2455–8. doi: 10.16368/j.issn.1674-8999.2021.11.508

[37] Gao HSX-P. Effect observation of electroacupuncture in the treatment of cognitive impairment after cerebral infarction. China Pract Med. (2023) 18: 41–4. doi: 10.14163/j.cnki.11-5547/r.2023.04.011

[38] Pan KPL Chen L. Effect of electroacupuncture Baihui and Sishencong acupoints combined with cognitive training on cognitive impairment after stroke. Reflexol Rehabil Med. (2023) 4:27–30.

[39] Wang HFX Chen Z. Clinical efficacy of electro-acupuncturing on Baihui and Zusanli points plus rehabilitation training on post-stroke cognitive impairment. Clin J Chin Med. (2017) 9:67–70. doi: 10.3969/j.issn.1674-7860.2017.05.035

[40] Feng BYX Wang S Liu B Zhao A Ding B. Effect of early scalp electroacupuncture combined with hyperbaric oxygen on cerebral edema and cognitive impairment in patients with hypertensive intracerebral hemorrhage. Mod J Integr Tradit Chin West Med. (2019) 28:413–23. Epub 20191110. doi: 10.3969/j.issn.1008-8849.2019.04.018

[41] Liu JFX. Clinical observation of treating cognitive impairment after stroke by electroacupuncture at Baihui and Shenting with cognitive rehabilitation training. China J Chin Med. (2013) 28:608–10. doi: 10.16368/j.issn.1674-8999.2013.04.020

[42] Han JZL Lan W. Clinical study of electroacupuncture combined with repetitive transcranial magnetic stimulation in treatment of mild cognitive impairment after ischemic stroke. Shandong J Tradit Chin Med. (2024) 43:160–6. doi: 10.16295/j.cnki.0257-358x.2024.02.010

[43] Run-li L. Clinical study of electric acupuncture Shenting and Baihui point on mild cognitive impairment after stroke. Clin Res Pract. (2017) 2:101–2. doi: 10.19347/j.cnki.2096-1413.201729049

[44] Song S-c ZJ-w Tian J-b . The clinical curative effect of acupuncture combined with medicine on stroke patients with cognitive impairment. J Emerg Tradit Chin Med. (2013) 22.

[45] Ma YHZ Wu Y Liu P Peng S. Effect of hyperbaric oxygen combined with electroacupuncture at Siguan acupoint on early cognitive impairment in stroke patients. Surg Res New Tech. (2018) 7:38–40. doi: 10.3969/j.issn.2095-378X.2018.01.011

[46] Yu B Xu J Zhu X. Therapeutic effects of scalp electroacupuncture on non-dementia vascular cognitive impairment and its effects on event-related potentials. World J Tradit Chin Med. (2020) 15

[47] Zhou Junying ZJ, Chen Bin Lyu Jianli. Effects of electroacupuncture at Baihui (Gv20) and Shenting (Gv24) on mild cognitive impairment after stroke. World J Tradit Chin Med (2019) 14:486–489. doi: 10.3969/j.issn.1673-7202.2019.02.050.

[48] Huang L Yin X Li W Cao Y Chen Y Lao L . Effects of acupuncture on vascular cognitive impairment with no dementia: a randomized controlled trial. J Alzheimer's Dis. (2021) 81:1391–401. doi: 10.3233/JAD-201353, 33935074 PMC8293636

[49] Li Zhanbiao GJ Fajing W Junyan M Zhenyan Z. Efficacy of repetitive transcranial magnetic stimulation combined with electroacupuncture in the treatment of mild cognitive impairment after ischemic stroke. Chin J Phys Med Rehabil. (2021) 43:628–30. doi: 10.3760/cma.j.issn.0254-1424.2021.07.011

[50] Lin Yanyue GX Huachao H Hanming W Shouyi L. The electric needle treatment brain obstructs the clinical research of cognizing the function damage. Chin J Med Guide. (2009) 11:2046–50. doi: 10.3969/j.issn.1009-0959.2009.12.029

[51] LY-q W Y-l dong TX . Effects of transcutaneous electrical acupoint stimulation in the treatment of post - stroke cognitive impairment: a randomized controlled clinical trial. Chin Gen Pract. (2017) 20. Avaialble online at: https://kns.cnki.net/kcms2/article/abstract?v=X84Xx1LLloLyLqDmU2XWr2Nyp1kQZmKLJbMZN_FUaZRra3EDZR_nxGjc_jAJTo8o41VZg996n8ZpA5xo06i7MmJcSz5gyCOvC5NplBX6ZHDSPzQFskX7Z9NJM0p-fT5PTiGnasUJgf4_NVoTJau-pk7kWSz3ow9P0Uts0BZm0KACb3HXGB7lGw==&uniplatform=NZKPT&language=CHS

[52] Zhao Yu ZH Ling Z. Observations on the efficacy of electric scalp acupuncture in treating mild cognitive impairment. J Sichuan Trad Chin Med. (2012) 30:112–4.

[53] Toyoda K Yoshimura S Nakai M Koga M Sasahara Y Sonoda K . Twenty-year change in severity and outcome of ischemic and hemorrhagic strokes. JAMA Neurol. (2022) 79:61–9. doi: 10.1001/jamaneurol.2021.4346, 34870689 PMC8649912

[54] El Husseini N Katzan IL Rost NS Blake ML Byun E Pendlebury ST . Cognitive impairment after ischemic and hemorrhagic stroke: a scientific statement from the American Heart Association/American Stroke Association. Stroke. (2023) 54:e272–91. doi: 10.1161/str.0000000000000430, 37125534 PMC12723706

[55] Bornstein NM Aronovich B Korczyn AD Shavit S Michaelson DM Chapman J. Antibodies to brain antigens following stroke. Neurology. (2001) 56:529–30. doi: 10.1212/wnl.56.4.529, 11222800

[56] Kim SH Jeong JH Lim JH Kim BK. Acupuncture using pattern-identification for the treatment of insomnia disorder: a systematic review and meta-analysis of randomized controlled trials. Integr Med Res. (2019) 8:216–26. doi: 10.1016/j.imr.2019.08.002, 31497504 PMC6718809

[57] Chen L Chen S Bai Y Zhang Y Li X Wang Y . Electroacupuncture improves cognitive impairment after ischemic stroke based on regulation of mitochondrial dynamics through Sirt1/Pgc-1α pathway. Brain Res. (2024) 1844:149139. doi: 10.1016/j.brainres.2024.149139, 39111521

[58] Zhang Y Gao S Lin L Zheng Y. Electroacupuncture therapy improves cognitive dysfunction after ischemic stroke in Sprague-Dawley rats by adjusting the Lncrna-Meg3/Mir-4640-3p axis. Ann Med Surg (Lond). (2025) 87:5512–21. doi: 10.1097/ms9.0000000000003628, 40901091 PMC12401418

[59] Lin B Wang M Chen X Chai L Ni J Huang J. Involvement of P2x7r-mediated microglia polarization and neuroinflammation in the response to electroacupuncture on post-stroke memory impairment. Brain Res Bull. (2024) 212:110967. doi: 10.1016/j.brainresbull.2024.110967, 38670470

[60] Chang QY Lin YW Hsieh CL. Acupuncture and Neuroregeneration in ischemic stroke. Neural Regen Res. (2018) 13:573–83. doi: 10.4103/1673-5374.230272, 29722298 PMC5950656

[61] Napadow V Dhond RP Kim J LaCount L Vangel M Harris RE . Brain encoding of acupuncture sensation--coupling on-line rating with Fmri. NeuroImage. (2009) 47:1055–65. doi: 10.1016/j.neuroimage.2009.05.079, 19500677 PMC2733781

[62] Chavez LM Huang SS MacDonald I Lin JG Lee YC Chen YH. Mechanisms of acupuncture therapy in ischemic stroke rehabilitation: a literature review of basic studies. Int J Mol Sci. (2017) 18. doi: 10.3390/ijms18112270, 29143805 PMC5713240

[63] Hopewell S Chan AW Collins GS Hróbjartsson A Moher D Schulz KF . Consort 2025 statement: updated guideline for reporting randomised trials. BMJ. (2025) 389:e081123. doi: 10.1136/bmj-2024-081123, 40228833 PMC11995449

[64] Yi Y Qu Y Lv S Zhang G Rong Y Li M. Comparative efficacy and safety of non-pharmacological interventions as adjunctive treatment for vascular dementia: a systematic review and network meta-analysis. Front Neurol. (2024) 15:1397088. doi: 10.3389/fneur.2024.1397088, 39070050 PMC11272661

[65] Li L Yang L Luo B Deng L Zhong Y Gan D . Acupuncture for post-stroke cognitive impairment: an overview of systematic reviews. Int J Gen Med. (2022) 15:7249–64. doi: 10.2147/ijgm.S376759, 36124104 PMC9482408

[66] Zhang J Ji C Zhai X Ren S Tong H. Global trends and hotspots in research on acupuncture for stroke: a bibliometric and visualization analysis. Eur J Med Res. (2023) 28:359. doi: 10.1186/s40001-023-01253-w, 37735698 PMC10512511

