# Supplementary material for: Efficacy and safety of electroacupuncture in the treatment of post-stroke cognitive impairment: a systematic review and meta-analysis
Source: Front Neurol. 2026 Jan 2;16:1715658. doi: 10.3389/fneur.2025.1715658 (PMC12807943; doi:10.3389/fneur.2025.1715658)
Supplement: Supplementary file 1 [file Data_Sheet_1.zip › 补充材料/S2 Material.pdf]

Search strategies for PubMed.

	Search number	Search Items
P	#1	Stroke [MeSH Terms]
	#2	(Ischemic stroke OR Cerebrovascular Stroke OR CVA OR Brain Vascular Accident OR Acute Stroke OR Apoplexy OR Cerebral stroke OR Cerebrovascular disease OR Cerebrovascular accident OR Cerebrovascular disorders OR Cerebral hemorrhage OR Hemorrhagic apoplexy OR hemorrhagic stroke OR Cerebral infarction OR Brain infarction) [Title/Abstract]
	#3	#1 OR #2
P	#4	Cognition [MeSH Terms]
	#5	(Cognitive Dysfunction OR Cognitive Impairments OR Mild Cognitive Impairment OR Mild Neurocognitive Disorder OR Cognitive Decline OR Mental Deterioration OR Cognitive deficit OR Cognitive deficit OR PSCI) [Title/Abstract]
	#6	#4 OR #5
I	#7	Electroacupuncture [MeSH Terms]
	#8	(electric acupuncture OR electrical acupuncture OR electrical acupoint stimulation) [Title/Abstract]
	#9	#7 OR #8
RCT	#10	randomized controlled trial [MeSH Terms]
	#11	(randomized OR placebo) [Title/Abstract]
	#12	#10 OR #11
	#13	#3 AND #6 AND #9 AND #12

Search strategies for Cochrane Library

	Search number	Search Items
P	#1	MeSH descriptor: [Stroke] explode all trees
	#2	(Apoplexy):ti,ab,kw OR (Cerebral stroke):ti,ab,kw OR (Cerebrovascular disease):ti,ab,kw OR (Cerebrovascular accident):ti,ab,kw OR (Cerebrovascular disorders):ti,ab,kw
	#3	(Cerebral hemorrhage):ti,ab,kw OR (Cerebral hemorrhage):ti,ab,kw OR (hemorrhagic stroke):ti,ab,kw OR (Cerebral infarction):ti,ab,kw OR (Brain infarction):ti,ab,kw
	#4	(Ischemic stroke):ti,ab,kw OR (Cerebrovascular Stroke):ti,ab,kw OR (CVA):ti,ab,kw OR (Brain Vascular Accident):ti,ab,kw OR (Acute Stroke):ti,ab,kw
	#5	#1 OR #2 OR #3 OR # 4
P	#6	MeSH descriptor: [Cognition] explode all trees
	#7	(Cognitive Dysfunction):ti,ab,kw OR (Cognitive Impairments):ti,ab,kw OR (Mild Cognitive Impairment):ti,ab,kw OR (Mild Neurocognitive Disorder):ti,ab,kw OR (Cognitive Decline):ti,ab,kw
	#8	(Mental Deterioration):ti,ab,kw OR (Cognitive deficit):ti,ab,kw OR (PSCI):ti,ab,kw
	#9	#6 OR #7 OR #8
I	#10	MeSH descriptor: [Electroacupuncture] explode all trees
	#11	(electric acupuncture):ti,ab,kw OR (electrical acupoint stimulation):ti,ab,kw OR (electrical acupuncture):ti,ab,kw OR (electronic acupuncture):ti,ab,kw OR (electro-acupuncture):ti,ab,kw
	#12	#10 OR #11
RCT	#13	MeSH descriptor: [Randomized Controlled Trial] explode all trees
	#14	(randomized):ti,ab,kw OR (placebo):ti,ab,kw OR (random):ti,ab,kw
	#15	#13 OR #14
	#16	#5 AND #9 AND #12 AND #15

Search strategies for Embase

	Search number	Search Items
P	#1	*cerebrovascular accident/exp
	#2	'cerebrovascular accident':ab,ti OR 'cerebrovascular apoplexy':ab,ti OR stroke:ab ti OR 'cerebrovascular stroke':ab, ti OR cvas:ab,ti OR strokes:ab, ti OR 'apoplexy, cerebrovascular:ab,ti OR 'vascular accident, brain':ab,ti OR 'brain vascular accident':ab,ti OR 'brain vascular accidents':ab,ti OR 'stroke, cerebrovascular':ab,ti OR apoplexy:ab,ti OR 'cerebral stroke' :ab,ti OR 'acute strokes':ab,ti
P	#3	'cognition/exp
	#4	'cognitive dysfunction*:ab,ti OR 'dysfunctions, cognitive':ab,ti OR 'dysfunction, cognitive':ab, ti OR 'cognitive impairments':ab,ti OR 'cognitive impairment:ab,ti OR 'mild cognitive impairment*':ab,ti OR 'cognitive impairment, mild':ab,ti OR mild neurocognitive disorder': ab, ti OR 'isorders, mild neurocognitive':ab,ti OR 'cognitive decline*:ab,ti OR 'deterioration, mental':ab,ti OR 'mental deterioration*:ab, ti
I	#5	'electroacupuncture /exp
	#6	'acupuncture, electric':ab,ti OR 'electric acupuncture':ab,ti OR 'electrical acupoint stimulation':ab,ti OR 'electrical acupuncture':ab,ti OR 'electro-acupuncture':ab,ti OR 'electrode acupuncture':ab,ti OR 'electronic acupuncture':ab,ti OR 'electroacupuncture' ab,ti 'electroacupuncture /exp
RCT	#7	'randomized controlled trial'/exp
	#8	'controlled trial, randomized':ab,ti OR 'randomised controlled study':ab,ti OR 'randomised controlled trial:ab,ti OR 'randomized controlled study':ab, ti OR 'trial, randomized controlled'':ab,ti OR 'randomized controlled trial':ab,ti OR placebo:ab,ti OR random:ab,ti
	#9	#1 OR #2
	#10	#3 OR #4
	#11	#5 OR #6
	#12	#7 OR #8
	#13	#9 AND #10 AND #11 AND #12

Search strategies for web of science

	Search number	Search Items
P	#1	TS= (Stroke OR Ischemic stroke OR Cerebrovascular Stroke OR CVA OR Brain Vascular Accident OR Acute Stroke OR Apoplexy OR Cerebral stroke OR Cerebrovascular disease OR Cerebrovascular accident OR Cerebrovascular disorders OR Cerebral hemorrhage OR Hemorrhagic apoplexy OR hemorrhagic stroke OR Cerebral infarction OR Brain infarction)
P	#2	TS=(Cognition OR Cognitive Dysfunction OR Cognitive Impairments OR Mild Cognitive Impairment OR Mild Neurocognitive Disorder OR Cognitive Decline OR Mental Deterioration OR Cognitive deficit OR Cognitive deficit OR PSCI)
I	#3	TS=(Electroacupuncture OR electric acupuncture OR electrical acupuncture OR electrical acupoint stimulation)
RCT	#4	TS=(randomized controlled trial OR randomized OR placebo)
	#5	#4 AND #3 AND #2 AND #1

Search strategies for CNKI

	Search number	Search Items
P	#1	(SU-脑卒中) OR (SU-中风) OR (SU-脑血管障碍) OR (SU-脑梗死) OR (SU-脑栓塞) OR(SU-脑出血) OR (SU-脑梗塞)
P	#2	(SU-认知障碍) OR (SU-认知功能障碍)
I	#3	(SU-电针) OR (SU-针灸) OR (SU-针刺)
RCT	#4	(AB-随机对照) OR (AB-随机) OR (AB-RCT)
	#5	#4 AND #3 AND #2 AND #1

English Translation of the CNKI Search Strategy

	Search number	Search Items
P	#1	(SU=Stroke) OR (SU=Apoplexy) OR (SU=Cerebrovascular Disorders) OR (SU=Cerebral Infarction) OR (SU=Brain Embolism) OR (SU=Cerebral Hemorrhage) OR (SU=Brain Infarction)
P	#2	(SU=Cognitive Dysfunction) OR (SU=Cognitive Impairment)
I	#3	(SU=Electroacupuncture) OR (SU=Acupuncture) OR (SU=Acupuncture Therapy)
RCT	#4	(AB=Randomized Controlled Trial) OR (AB=Randomized) OR (AB=RCT)
	#5	#4 AND #3 AND #2 AND #1

Search strategies for VIP

	Search number	Search Items
P	#1	(SU-脑卒中) OR (SU-中风) OR (SU-脑血管障碍) OR (SU-脑梗死) OR (SU-脑栓塞) OR(SU-脑出血) OR (SU-脑梗塞)
P	#2	(SU-认知障碍) OR (SU-认知功能障碍)
I	#3	(SU-电针) OR (SU-针灸) OR (SU-针刺)
RCT	#4	(AB-随机对照) OR (AB-随机) OR (AB-RCT)
	#5	#4 AND #3 AND #2 AND #1

English Translation of the VIP Search Strategy

	Search number	Search Items
P	#1	(SU=Stroke) OR (SU=Apoplexy) OR (SU=Cerebrovascular Disorders) OR (SU=Cerebral Infarction) OR (SU=Brain Embolism) OR (SU=Cerebral Hemorrhage) OR (SU=Brain Infarction)
P	#2	(SU=Cognitive Dysfunction) OR (SU=Cognitive Impairment)
I	#3	(SU=Electroacupuncture) OR (SU=Acupuncture) OR (SU=Acupuncture Therapy)
RCT	#4	(AB=Randomized Controlled Trial) OR (AB=Randomized) OR (AB=RCT)
	#5	#4 AND #3 AND #2 AND #1

Search strategies for Wanfang Data

	Search number	Search Items
P	#1	(SU-脑卒中) OR (SU-中风) OR (SU-脑血管障碍) OR (SU-脑梗死) OR (SU-脑栓塞) OR(SU-脑出血) OR (SU-脑梗塞)
P	#2	(SU-认知障碍) OR (SU-认知功能障碍)
I	#3	(SU-电针) OR (SU-针灸) OR (SU-针刺)
RCT	#4	(AB-随机对照) OR (AB-随机) OR (AB-RCT)
	#5	#4 AND #3 AND #2 AND #1

English Translation of the Wanfang Data Search Strategy

	Search number	Search Items
P	#1	(SU=Stroke) OR (SU=Apoplexy) OR (SU=Cerebrovascular Disorders) OR (SU=Cerebral Infarction) OR (SU=Brain Embolism) OR (SU=Cerebral Hemorrhage) OR (SU=Brain Infarction)
P	#2	(SU=Cognitive Dysfunction) OR (SU=Cognitive Impairment)
I	#3	(SU=Electroacupuncture) OR (SU=Acupuncture) OR (SU=Acupuncture Therapy)
RCT	#4	(AB=Randomized Controlled Trial) OR (AB=Randomized) OR (AB=RCT)

	#5	#4 AND #3 AND #2 AND #1

Search strategies for SinoMed

	Search number	Search Items
P	#1	(SU-脑卒中) OR (SU-中风) OR (SU-脑血管障碍) OR (SU-脑梗死) OR (SU-脑栓塞) OR(SU-脑出血) OR (SU-脑梗塞)
P	#2	(SU-认知障碍) OR (SU-认知功能障碍)
I	#3	(SU-电针) OR (SU-针灸) OR (SU-针刺)
RCT	#4	(AB-随机对照) OR (AB-随机) OR (AB-RCT)
	#5	#4 AND #3 AND #2 AND #1

English Translation of the SinoMed Search Strategy

	Search number	Search Items
P	#1	(SU=Stroke) OR (SU=Apoplexy) OR (SU=Cerebrovascular Disorders) OR (SU=Cerebral Infarction) OR (SU=Brain Embolism) OR (SU=Cerebral Hemorrhage) OR (SU=Brain Infarction)
P	#2	(SU=Cognitive Dysfunction) OR (SU=Cognitive Impairment)
I	#3	(SU=Electroacupuncture) OR (SU=Acupuncture) OR (SU=Acupuncture Therapy)
RCT	#4	(AB=Randomized Controlled Trial) OR (AB=Randomized) OR (AB=RCT)
	#5	#4 AND #3 AND #2 AND #1
