# Supplementary material for: Efficacy and safety of electroacupuncture in the treatment of post-stroke cognitive impairment: a systematic review and meta-analysis
Source: Front Neurol. 2026 Jan 2;16:1715658. doi: 10.3389/fneur.2025.1715658 (PMC12807943; doi:10.3389/fneur.2025.1715658)
Supplement: Supplementary file 1 [file Data_Sheet_1.zip › 补充材料/S3 Supplementary figure.pdf]

Study or Subgroup	Treatment Mean	Treatment SD	Treatment Total	Control Mean	Control SD	Control Total	Weight(%)	Mean Difference IV, Random, 95% CI
**2 weeks**								
Liu 2017	19.93	1.17	32	14.03	0.98	32	6.5	5.90 [5.37; 6.43]
**4 weeks**								
Liu 2013	19.84	1.25	25	13.52	1.01	25	6.4	6.32 [5.69; 6.95]
Pan 2023	25.76	2.53	45	23.59	3.72	45	5.8	2.17 [0.86; 3.48]
Song 2013	26.53	2.45	60	24.62	2.54	60	6.2	1.91 [1.02; 2.80]
Zhou 2019	24.89	4.28	60	20.12	4.12	60	5.6	4.77 [3.27; 6.27]
Chen 2013	21.50	2.13	30	20.10	0.92	30	6.3	1.40 [0.57; 2.23]
Zhou 2013	28.10	0.60	30	27.12	1.75	30	6.4	0.98 [0.32; 1.64]
Lin 2009	24.12	3.86	28	23.98	4.35	21	4.5	0.14 [-2.21; 2.49]
Ma 2018	24.12	3.85	30	20.17	2.65	30	5.4	3.95 [2.28; 5.62]
Li 2023	25.86	2.45	30	22.61	2.21	30	5.9	3.25 [2.07; 4.43]
Li 2021	26.80	1.30	30	25.70	1.10	30	6.4	1.10 [0.49; 1.71]
**Total(95% CI)**			**368**			**361**	**59.0**	**2.63 [1.42; 3.83]**
Heterogeneity: $Tau^2 = 3.4067$; $Chi^2 = 206.84$, df = 9 (P < 0.0001); $I^2 = 95.6\%$								
**6 weeks**								
Gao 2023	22.97	1.86	45	21.17	1.63	45	6.4	1.80 [1.08; 2.52]
Yu 2020	26.45	3.11	37	23.56	2.78	35	5.7	2.89 [1.53; 4.25]
**Total(95% CI)**			**82**			**80**	**12.1**	**2.19 [1.16; 3.21]**
Heterogeneity: $Tau^2 = 0.2850$; $Chi^2 = 1.92$, df = 1 (P = 0.1656); $I^2 = 48\%$								
**8 weeks**								
Wang 2017	24.50	3.48	30	22.80	2.90	30	5.4	1.70 [0.08; 3.32]
Sun 2017	23.17	4.31	30	19.69	3.97	30	4.8	3.48 [1.38; 5.58]
Zhao 2012	27.93	1.41	26	27.43	1.38	26	6.3	0.50 [-0.26; 1.26]
Li 2021	26.41	3.47	60	24.40	3.85	60	5.8	2.01 [0.70; 3.32]
**Total(95% CI)**			**146**			**146**	**22.4**	**1.69 [0.51; 2.87]**
Heterogeneity: $Tau^2 = 0.9207$; $Chi^2 = 9.64$, df = 3 (P = 0.0218); $I^2 = 68.9\%$								
**Total(95% CI)**			**628**			**619**	**100.0**	**2.62 [1.74; 3.51]**

Heterogeneity: $Tau^2 = 3.0555$; $Chi^2 = 385.80$, df = 16 (P < 0.0001); $I^2 = 95.9\%$
Test for overall effect: Z = 5.82 (P < 0.0001)
Test for subgroup differences: $Chi^2 = 77.61$, df = 3 (P < 0.0001)

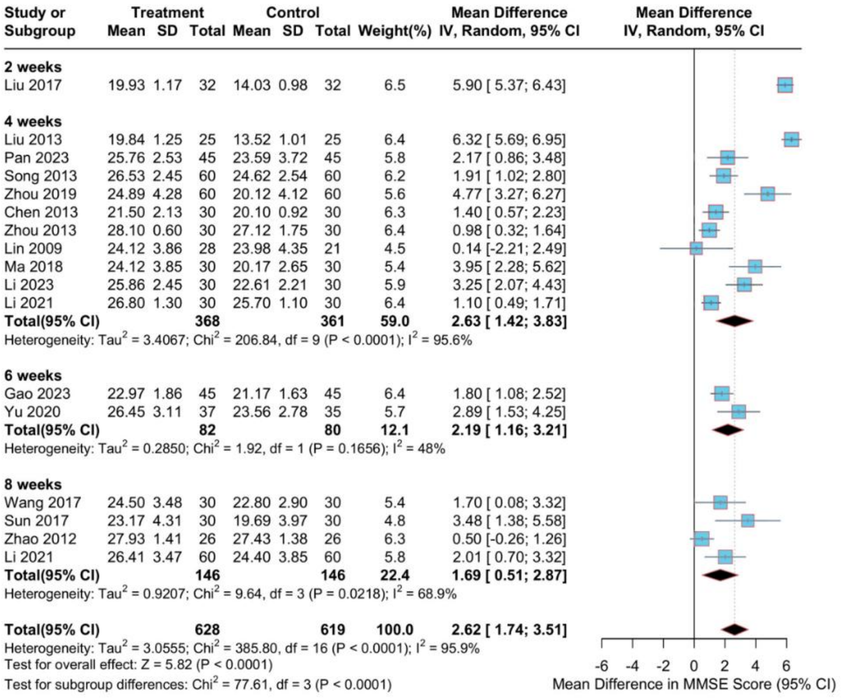

Figure 1: Subgroup Analysis of MMSE by treatment duration

Study or Subgroup	Treatment Mean	Treatment SD	Treatment Total	Control Mean	Control SD	Control Total	Weight(%)	Mean Difference IV, Random, 95% CI
**PSCI Severity = mild**								
Liu 2017	19.93	1.17	32	14.03	0.98	32	6.5	5.90 [5.37; 6.43]
Song 2013	26.53	2.45	60	24.62	2.54	60	6.2	1.91 [1.02; 2.80]
Zhou 2013	28.10	0.60	30	27.12	1.75	30	6.4	0.98 [0.32; 1.64]
Ma 2018	24.12	3.85	30	20.17	2.65	30	5.4	3.95 [2.28; 5.62]
Gao 2023	22.97	1.86	45	21.17	1.63	45	6.4	1.80 [1.08; 2.52]
Yu 2020	26.45	3.11	37	23.56	2.78	35	5.7	2.89 [1.53; 4.25]
Li Z 2021	26.80	1.30	30	25.70	1.10	30	6.4	1.10 [0.49; 1.71]
Zhao 2012	27.93	1.41	26	27.43	1.38	26	6.3	0.50 [-0.26; 1.26]
Li 2021	26.41	3.47	60	24.40	3.85	60	5.8	2.01 [0.70; 3.32]
**Total(95% CI)**			**350**			**348**	**55.2**	**2.31 [1.17; 3.46]**
Heterogeneity: $Tau^2 = 2.8095$; $Chi^2 = 235.07$, df = 8 (P < 0.0001); $I^2 = 96.6\%$								
**PSCI Severity = moderate**								
Liu 2013	19.84	1.25	25	13.52	1.01	25	6.4	6.32 [5.69; 6.95]
Wang 2017	24.50	3.48	30	22.80	2.90	30	5.4	1.70 [0.08; 3.32]
Pan 2023	25.76	2.53	45	23.59	3.72	45	5.8	2.17 [0.86; 3.48]
Zhou 2019	24.89	4.28	60	20.12	4.12	60	5.6	4.77 [3.27; 6.27]
Sun 2017	23.17	4.31	30	19.69	3.97	30	4.8	3.48 [1.38; 5.58]
Chen 2013	21.50	2.13	30	20.10	0.92	30	6.3	1.40 [0.57; 2.23]
Lin 2009	24.12	3.86	28	23.98	4.35	21	4.5	0.14 [-2.21; 2.49]
Li 2023	25.86	2.45	30	22.61	2.21	30	5.9	3.25 [2.07; 4.43]
**Total(95% CI)**			**278**			**271**	**44.8**	**2.99 [1.59; 4.39]**
Heterogeneity: $Tau^2 = 3.5055$; $Chi^2 = 117.69$, df = 7 (P < 0.0001); $I^2 = 94.1\%$								
**Total(95% CI)**			**628**			**619**	**100.0**	**2.62 [1.74; 3.51]**

Heterogeneity: $Tau^2 = 3.0555$; $Chi^2 = 385.80$, df = 16 (P < 0.0001); $I^2 = 95.9\%$
Test for overall effect: Z = 5.82 (P < 0.0001)
Test for subgroup differences: $Chi^2 = 0.55$, df = 1 (P = 0.4599)

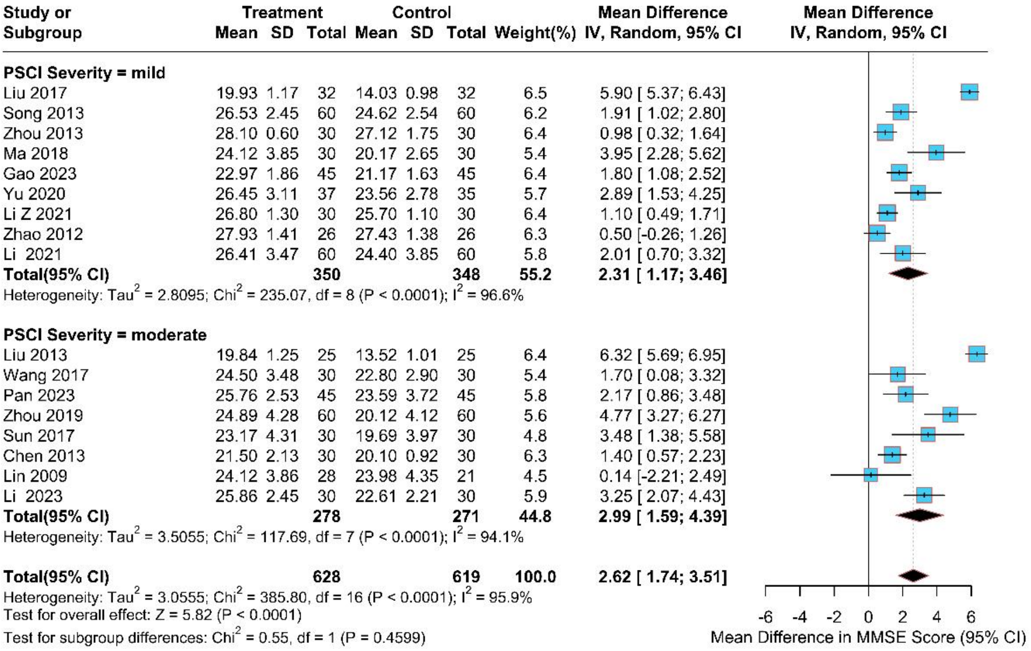

Figure 2: Subgroup Analysis of MMSE by PSCI Severity

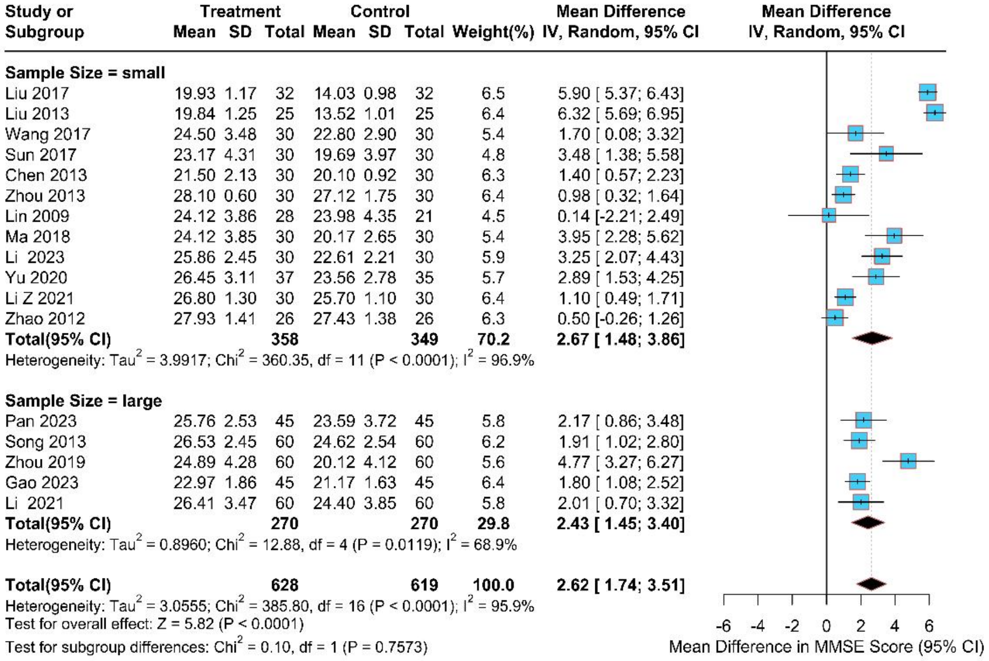

Figure 3: Subgroup Analysis of MMSE by Sample Size

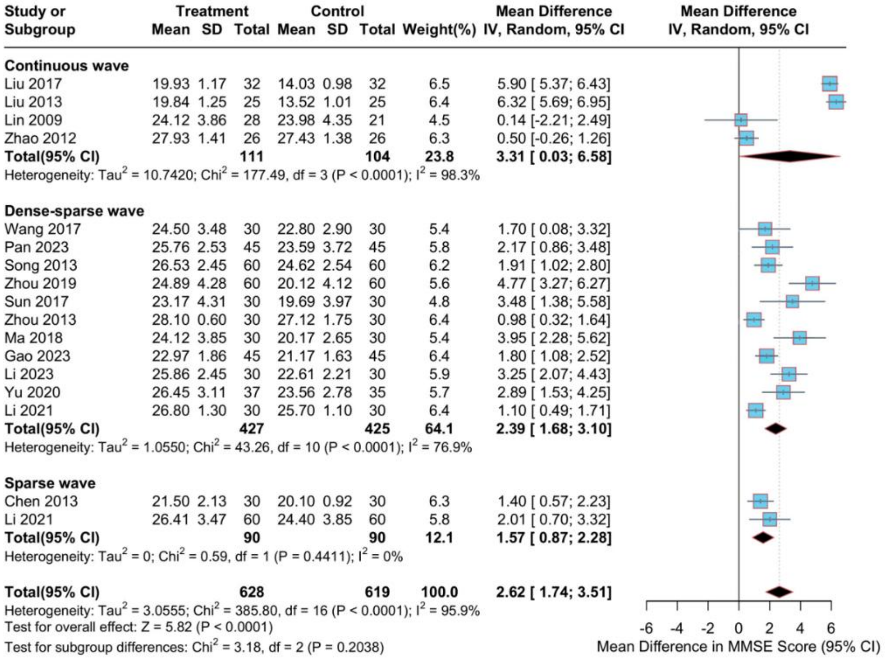

Figure 4: Subgroup Analysis of MMSE by Electroacupuncture Waveform Type

Study or Subgroup	Treatment Mean	Treatment SD	Treatment Total	Control Mean	Control SD	Control Total	Weight(%)	Mean Difference IV, Random, 95% CI
**Acupuncture.Location = Head**								
Liu 2017	19.93	1.17	32	14.03	0.98	32	6.5	5.90 [5.37; 6.43]
Liu 2013	19.84	1.25	25	13.52	1.01	25	6.4	6.32 [5.69; 6.95]
Pan 2023	25.76	2.53	45	23.59	3.72	45	5.8	2.17 [0.86; 3.48]
Song 2013	26.53	2.45	60	24.62	2.54	60	6.2	1.91 [1.02; 2.80]
Zhou 2019	24.89	4.28	60	20.12	4.12	60	5.6	4.77 [3.27; 6.27]
Yu 2020	26.45	3.11	37	23.56	2.78	35	5.7	2.89 [1.53; 4.25]
Zhao 2012	27.93	1.41	26	27.43	1.38	26	6.3	0.50 [-0.26; 1.26]
**Total(95% CI)**			**285**			**283**	**42.6**	**3.51 [1.84; 5.18]**
Heterogeneity: $Tau^2 = 4.7799$; $Chi^2 = 213.1$, df = 6 (P < 0.0001); $I^2 = 97.2\%$								
**Acupuncture.Location = Head + Limbs**								
Wang 2017	24.50	3.48	30	22.80	2.90	30	5.4	1.70 [0.08; 3.32]
Sun 2017	23.17	4.31	30	19.69	3.97	30	4.8	3.48 [1.38; 5.58]
Chen 2013	21.50	2.13	30	20.10	0.92	30	6.3	1.40 [0.57; 2.23]
Lin 2009	24.12	3.86	28	23.98	4.35	21	4.5	0.14 [-2.21; 2.49]
Gao 2023	22.97	1.86	45	21.17	1.63	45	6.4	1.80 [1.08; 2.52]
Li 2023	25.86	2.45	30	22.61	2.21	30	5.9	3.25 [2.07; 4.43]
Li Z 2021	26.80	1.30	30	25.70	1.10	30	6.4	1.10 [0.49; 1.71]
Li 2021	26.41	3.47	60	24.40	3.85	60	5.8	2.01 [0.70; 3.32]
**Total(95% CI)**			**283**			**276**	**45.6**	**1.81 [1.22; 2.40]**
Heterogeneity: $Tau^2 = 0.3493$; $Chi^2 = 15.51$, df = 7 (P = 0.0300); $I^2 = 54.9\%$								
**Acupuncture.Location = Limbs**								
Zhou 2013	28.10	0.60	30	27.12	1.75	30	6.4	0.98 [0.32; 1.64]
Ma 2018	24.12	3.85	30	20.17	2.65	30	5.4	3.95 [2.28; 5.62]
**Total(95% CI)**			**60**			**60**	**11.8**	**2.36 [-0.54; 5.27]**
Heterogeneity: $Tau^2 = 3.9893$; $Chi^2 = 10.47$, df = 1 (P = 0.0012); $I^2 = 90.5\%$								
**Total(95% CI)**			**628**			**619**	**100.0**	**2.62 [1.74; 3.51]**

Heterogeneity: $Tau^2 = 3.0555$; $Chi^2 = 385.80$, df = 16 (P < 0.0001); $I^2 = 95.9\%$
Test for overall effect: Z = 5.82 (P < 0.0001)
Test for subgroup differences: $Chi^2 = 3.62$, df = 2 (P = 0.1640)

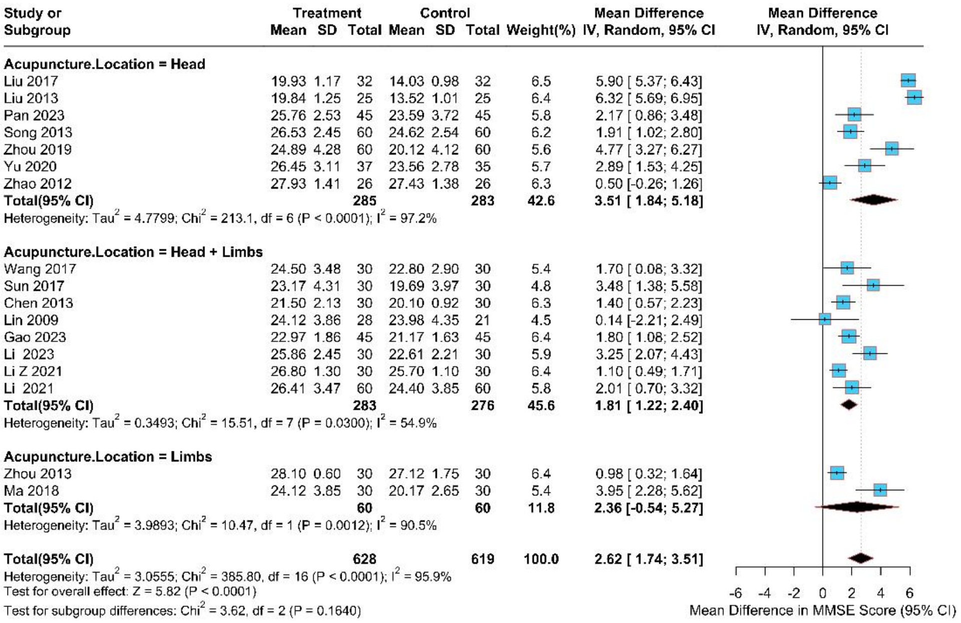

Figure 5:Subgroup Analysis of MMSE by acupuncture location

Study or Subgroup	Treatment Mean	Treatment SD	Treatment Total	Control Mean	Control SD	Control Total	Weight(%)	Mean Difference IV, Random, 95% CI
**Control group intervention = Cognitive training**								
Liu 2017	19.93	1.17	32	14.03	0.98	32	6.5	5.90 [5.37; 6.43]
Liu 2013	19.84	1.25	25	13.52	1.01	25	6.4	6.32 [5.69; 6.95]
Wang 2017	24.50	3.48	30	22.80	2.90	30	5.4	1.70 [0.08; 3.32]
Pan 2023	25.76	2.53	45	23.59	3.72	45	5.8	2.17 [0.86; 3.48]
Zhou 2019	24.89	4.28	60	20.12	4.12	60	5.6	4.77 [3.27; 6.27]
Zhou 2013	28.10	0.60	30	27.12	1.75	30	6.4	0.98 [0.32; 1.64]
Gao 2023	22.97	1.86	45	21.17	1.63	45	6.4	1.80 [1.08; 2.52]
Li 2023	25.86	2.45	30	22.61	2.21	30	5.9	3.25 [2.07; 4.43]
**Total(95% CI)**			**297**			**297**	**48.4**	**3.39 [1.93; 4.84]**
Heterogeneity: $Tau^2 = 4.1280$; $Chi^2 = 234.88$, df = 7 (P < 0.0001); $I^2 = 97\%$								
**Control group intervention = Western Medicine**								
Song 2013	26.53	2.45	60	24.62	2.54	60	6.2	1.91 [1.02; 2.80]
Sun 2017	23.17	4.31	30	19.69	3.97	30	4.8	3.48 [1.38; 5.58]
Yu 2020	26.45	3.11	37	23.56	2.78	35	5.7	2.89 [1.53; 4.25]
Li Z 2021	26.80	1.30	30	25.70	1.10	30	6.4	1.10 [0.49; 1.71]
Zhao 2012	27.93	1.41	26	27.43	1.38	26	6.3	0.50 [-0.26; 1.26]
**Total(95% CI)**			**183**			**181**	**29.6**	**1.75 [0.77; 2.72]**
Heterogeneity: $Tau^2 = 0.9166$; $Chi^2 = 15.85$, df = 4 (P = 0.0032); $I^2 = 74.8\%$								
**Control group intervention = Acupuncture Therapy**								
Chen 2013	21.50	2.13	30	20.10	0.92	30	6.3	1.40 [0.57; 2.23]
Lin 2009	24.12	3.86	28	23.98	4.35	21	4.5	0.14 [-2.21; 2.49]
Li 2021	26.41	3.47	60	24.40	3.85	60	5.8	2.01 [0.70; 3.32]
**Total(95% CI)**			**118**			**111**	**16.6**	**1.46 [0.78; 2.13]**
Heterogeneity: $Tau^2 = < 0.0001$; $Chi^2 = 1.91$, df = 2 (P = 0.3846); $I^2 = 0\%$								
**Control group intervention = Hyperbaric oxygen**								
Ma 2018	24.12	3.85	30	20.17	2.65	30	5.4	3.95 [2.28; 5.62]
**Total(95% CI)**			**628**			**619**	**100.0**	**2.62 [1.74; 3.51]**

Heterogeneity: $Tau^2 = 3.0555$; $Chi^2 = 385.80$, df = 16 (P < 0.0001); $I^2 = 95.9\%$
Test for overall effect: Z = 5.82 (P < 0.0001)
Test for subgroup differences: $Chi^2 = 11.45$, df = 3 (P = 0.0095)

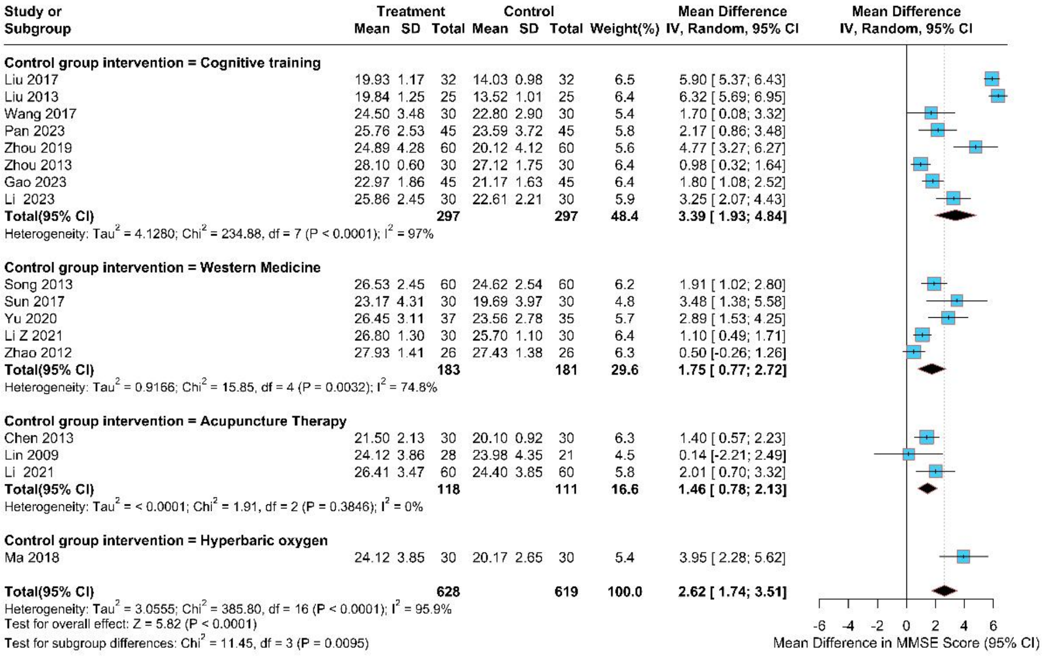

Figure 6:Subgroup Analysis of MMSE by control group intervention

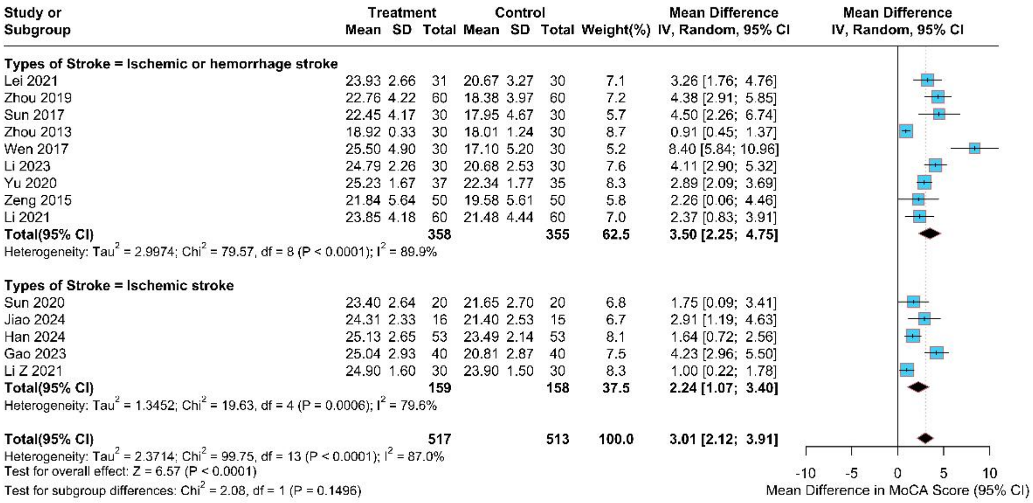

Figure 7: Subgroup Analysis of MoCA by Stroke Type

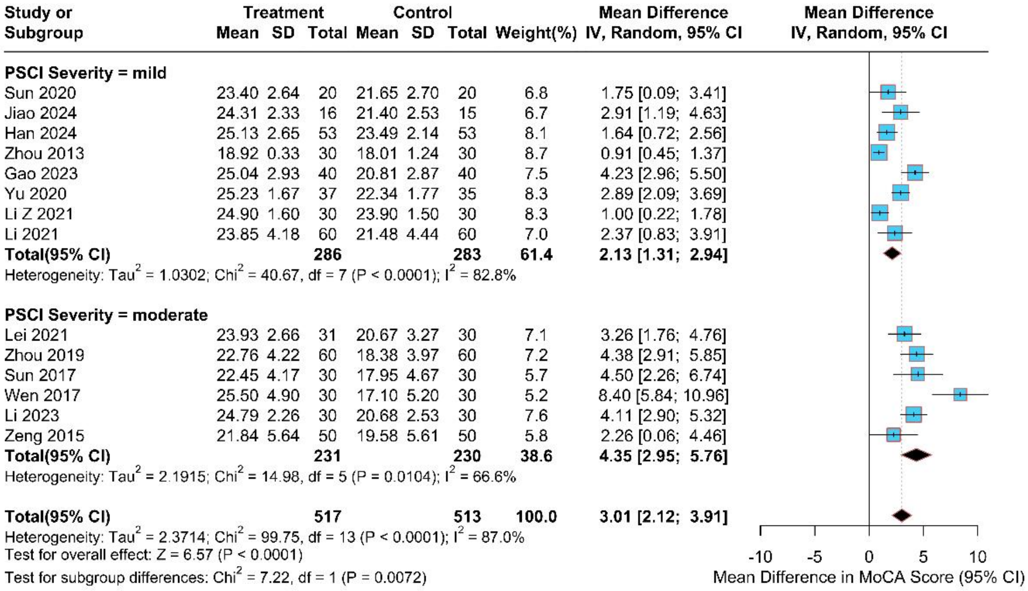

Figure 8: Subgroup Analysis of MoCA by PSCI Severity

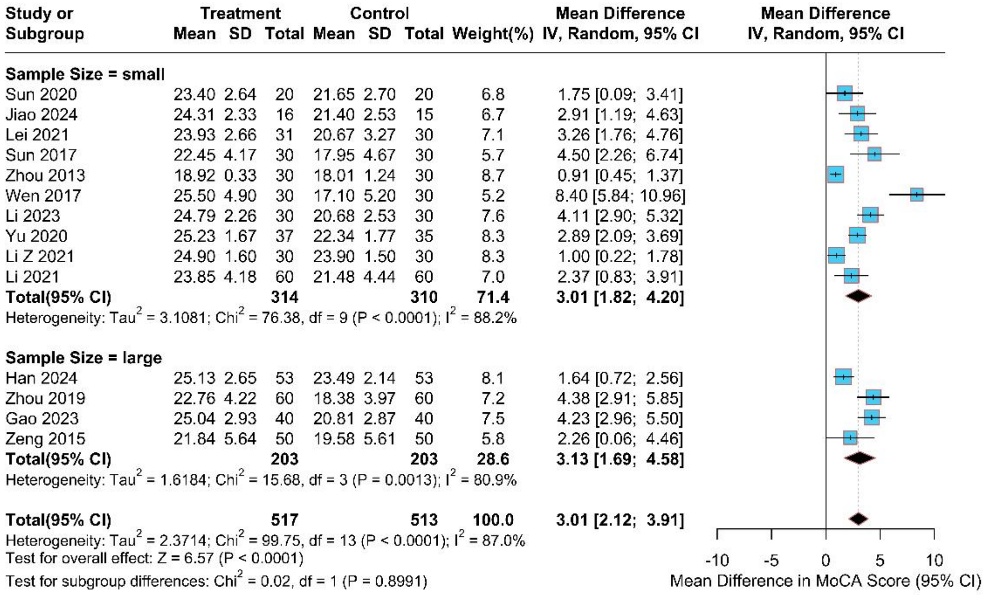

Figure 9: Subgroup Analysis of MoCA by Sample Size

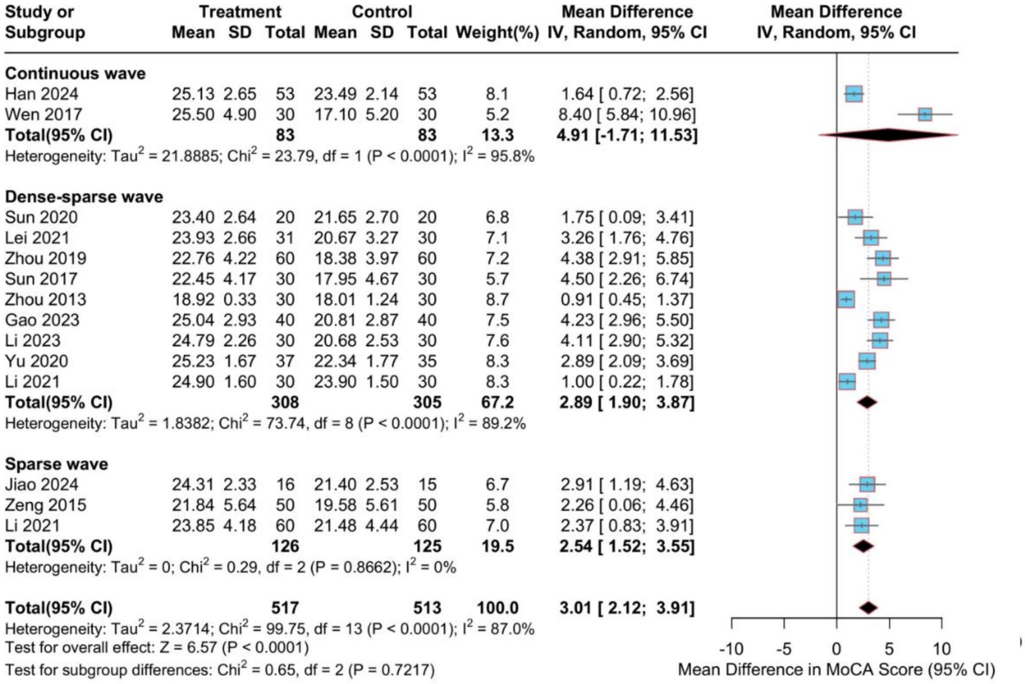

Figure 10: Subgroup Analysis of MoCA by Electroacupuncture Waveform Type

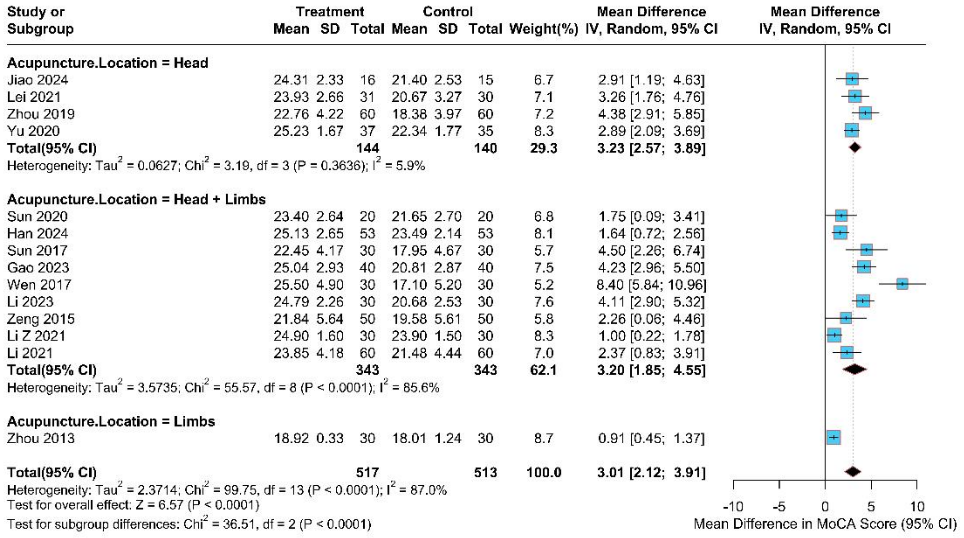

Figure 11:Subgroup Analysis of MoCA by acupuncture location

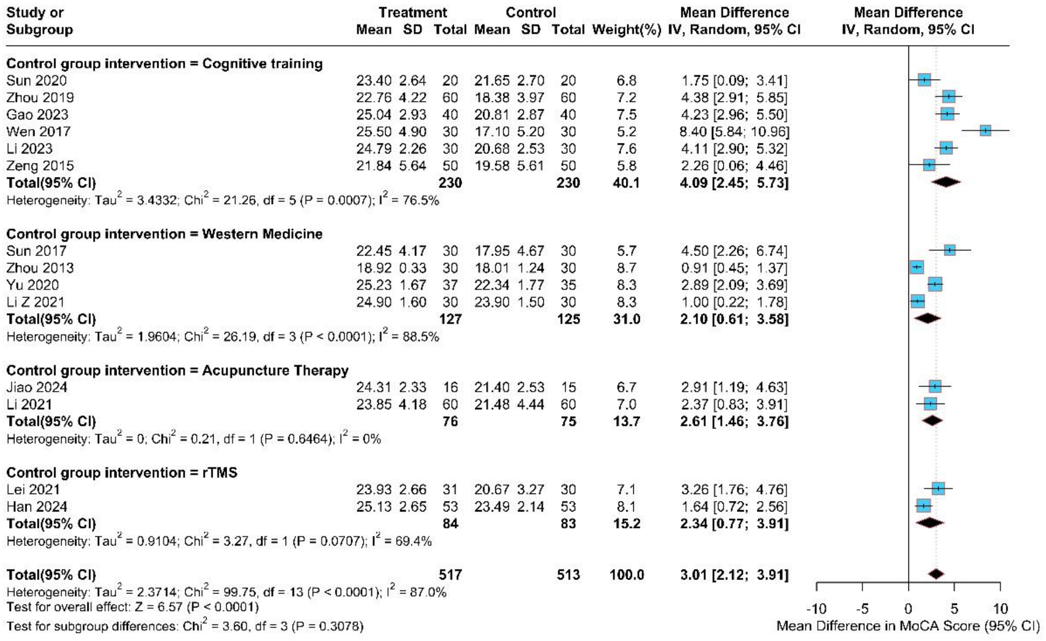

Figure 12:Subgroup Analysis of MoCA by control group intervention

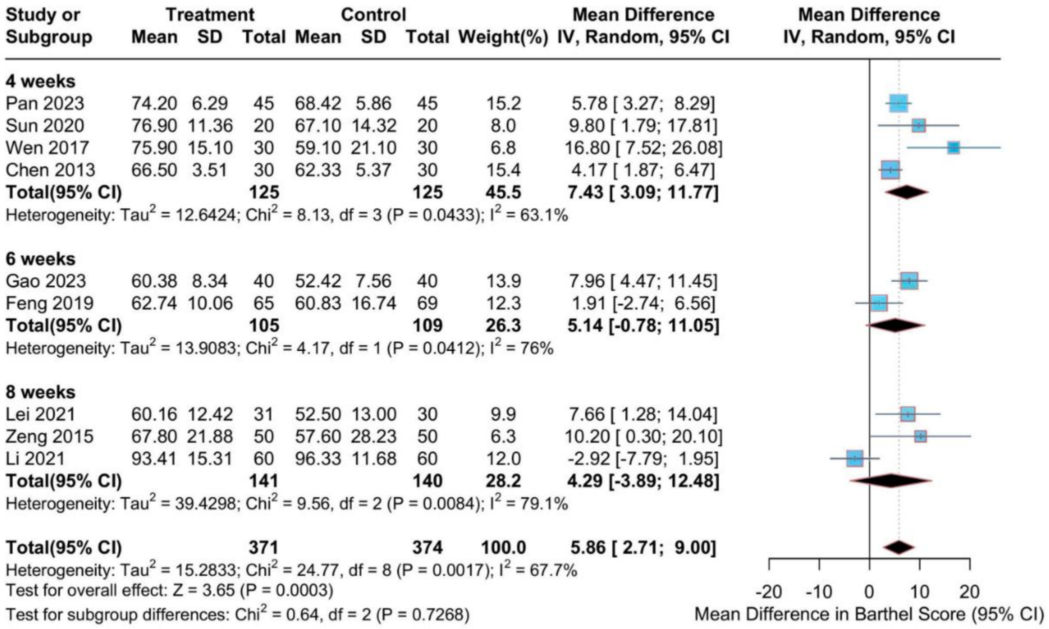

Figure 13: Subgroup Analysis of Barthel by treatment duration

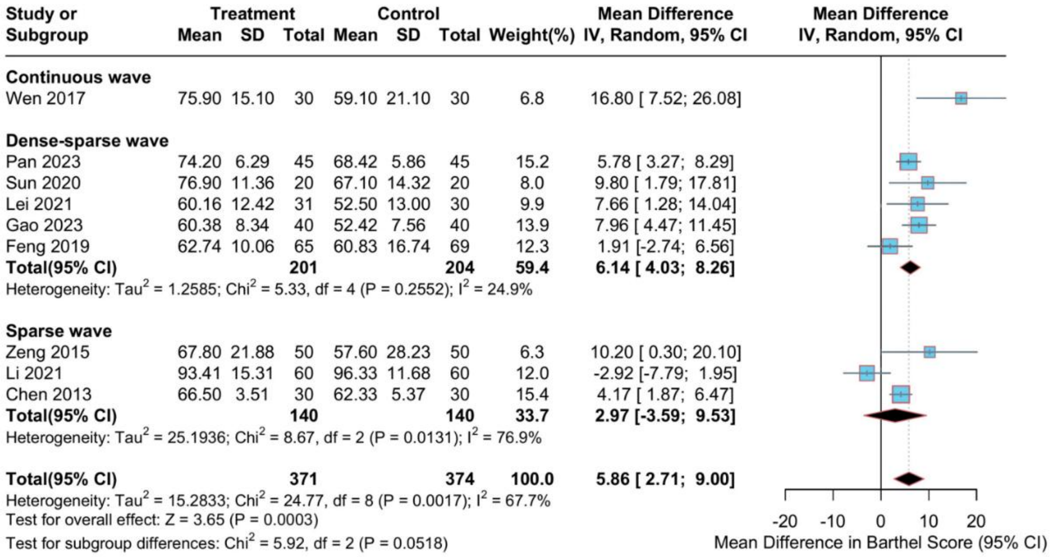

Figure 14: Subgroup Analysis of Barthel by Electroacupuncture Waveform Type

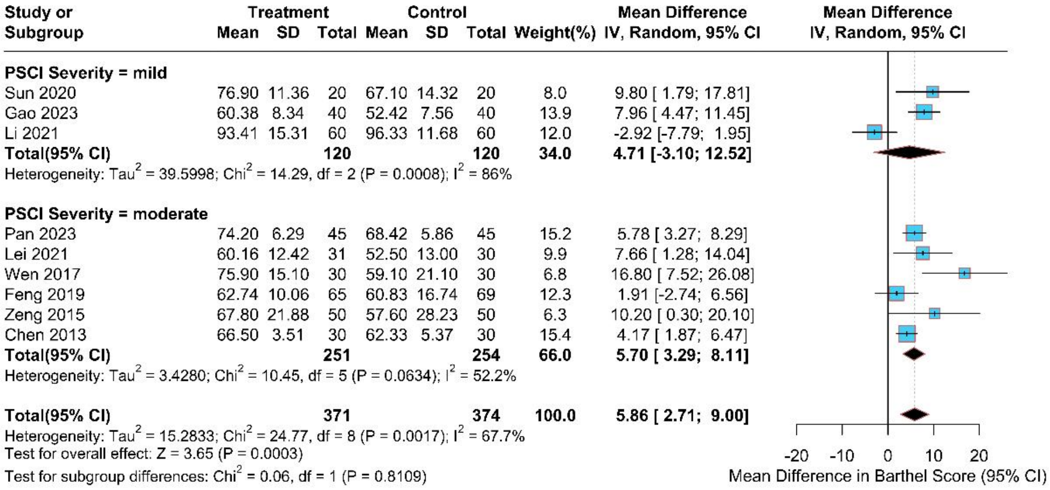

Figure 15: Subgroup Analysis of Barthel by PSCI Severity

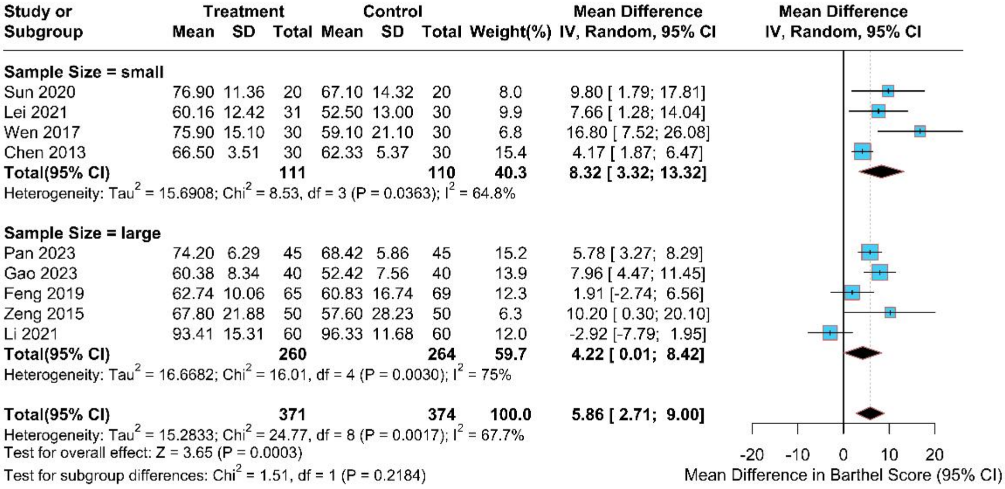

Figure 16: Subgroup Analysis of Barthel by Sample Size

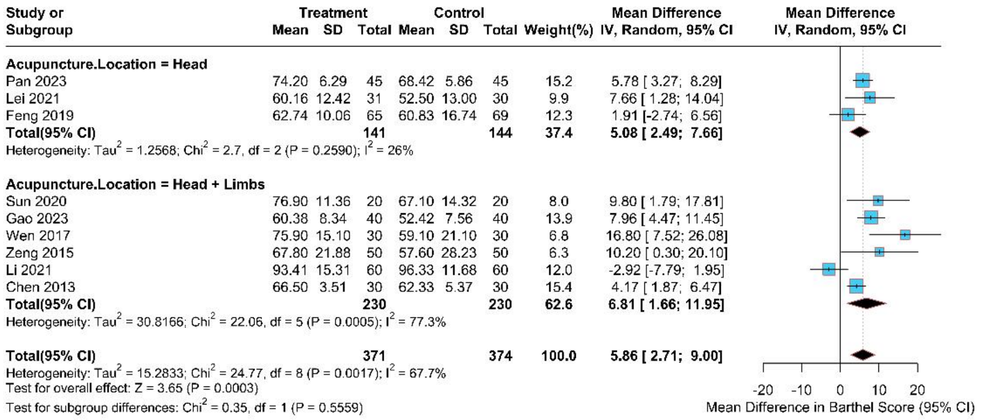

Figure 17:Subgroup Analysis of Barthel by acupuncture location

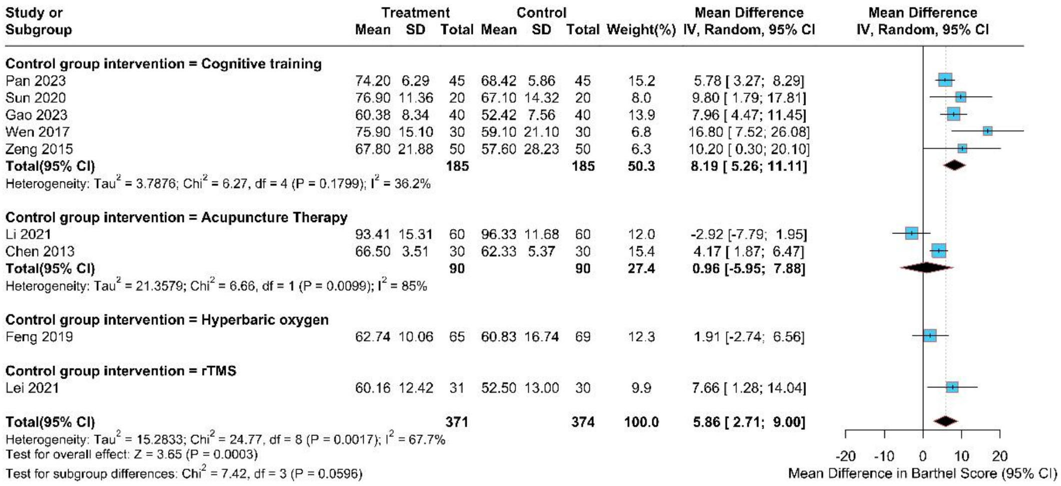

Figure 18:Subgroup Analysis of Barthel by control group intervention
